# The Bayesian Inference of Pareto Models Based on Information Geometry

**DOI:** 10.3390/e23010045

**Published:** 2020-12-30

**Authors:** Fupeng Sun, Yueqi Cao, Shiqiang Zhang, Huafei Sun

**Affiliations:** School of Mathematics and Statistics, Beijing Institute of Technology, Beijing 100081, China; fupeng_sun@bit.edu.cn (F.S.); yueqi_cao@bit.edu.cn (Y.C.); shiqiang@bit.edu.cn (S.Z.)

**Keywords:** Bayesian inference, Pareto two-parameter model, Jeffreys prior, mean geodesic estimation, Al-Bayyati’s loss function, 62C10, 62F15

## Abstract

Bayesian methods have been rapidly developed due to the important role of explicable causality in practical problems. We develope geometric approaches to Bayesian inference of Pareto models, and give an application to the analysis of sea clutter. For Pareto two-parameter model, we show the non-existence of *α*-parallel prior in general, hence we adopt Jeffreys prior to deal with the Bayesian inference. Considering geodesic distance as the loss function, an estimation in the sense of minimal mean geodesic distance is obtained. Meanwhile, by involving Al-Bayyati’s loss function we gain a new class of Bayesian estimations. In the simulation, for sea clutter, we adopt Pareto model to acquire various types of parameter estimations and the posterior prediction results. Simulation results show the advantages of the Bayesian estimations proposed and the posterior prediction.

## 1. Introduction

Geometric method plays an important role in Bayesian statistics. At present, there are two main ways to study Bayesian inference through geometric methods. An idea is to regard the prior distribution, the probability distribution of the statistical model and the posterior distribution as the vectors in Hilbert space $L^{2}\left(\Theta \right)$. The research is then carried out through the geometric properties of Hilbert space. M. de Carvalho [1] used the cosine of the angle between vectors to study their relationship with each other, where the cosine of priors represents coherency of opinions of experts, the cosine of prior and probability density represents prior-data agreement and the cosine of prior and posterior represents sensitivity of the posterior to the prior specification. Furthermore, M. de Carvalho used Monte Carlo Markov Chain to give an estimation of the cosine value for further analysis. R. Kulhavy [2] viewed statistical inference as an approximation of the empirical density rather than an estimation of a true density, and built a model by analyzing the trace of orthogonal projection of conditional empirical distributions onto the model manifold. He also used Kerridge inaccuracy as a generalized empirical error. Kerridge inaccuracy is a generalization of Shannon entropy. It is used to measure the difference between observed distribution $Q=(q_{1},\cdots ,q_{n})$ and true distribution $P=(p_{1},\cdots ,p_{n})$, which is defined by $I\left(P,Q\right)=-\sum_{i}p_{i}\log\left(q_{i}\right)$. The advantage of this idea is providing a unified treatment to all pieces of Bayesian theorem. However, the finite parameter space measure is required.

Another idea is to give the statistical manifolds Riemannian metrics. J.A. Hartigan [3,4,5] proposed a reparametrization invariance prior-α-parallel prior and later J. Takeuchi and S. Amari [6,7] clarified an interesting connection between the information geometrical properties of the statistical model and the existence of the α-parallel prior. α-parallel prior, as an uninformed prior, is invariant under the coordinate transformation and can well reflect the intrinsic properties of the model. It is worth noting that the general α-parallel prior does not always exist, but 0-parallel prior, Jeffreys prior, always exists. After obtaining the corresponding prior, subsequent Bayesian inference and prediction can be carried out. J. Takeuchi and S. Amari studied the asymptotic properties of minimum description length (MDL) estimation and projected Bayesian estimation of general exponential families, and extended it to curve exponential families. The differential geometry of curved exponential families are given by S. Amari and M. Kumon [8,9].

In many confidential fields, historical data is difficult to obtain. Hence one of the advantages of the above idea is giving prior a good theoretical basis. Besides, this idea can illustrate the geometric meaning of the common Bayesian estimations and provide an estimation in the sense of minimal mean geodesic distance. In the application of target detection, maritime radar performance is often seriously interfered and suppressed by sea clutter, especially for the detection of weak targets on the sea. Therefore, whether sea clutter can be effectively suppressed is the key factor to improve the performance of maritime radar systems. Thus the study of sea clutter is of vital importance [10]. With the development of radar hardware technology, the statistical distribution histogram of radar sea clutter appears long “trailing tail”, which is manifested as frequent sea peak phenomenon, and the amplitude distribution of clutter seriously deviates from the Rayleigh distribution proposed before. In order to solve this problem, an improved Rayleigh model-compound Gaussian model is proposed [11]. The compound Gaussian model successfully illustrates the formation mechanism of sea clutter, and is more successful than the single point probability distribution model in terms of data fitting. In the compound Gaussian model, *K* distribution and Pareto distribution are two typical representatives. When the structural component is Gamma distribution, the compound Gaussian model is *K* distribution. When the structural component is inverse Gamma distribution, the compound Gaussian model is Pareto distribution. In 2010, M. Farshchian and F.L. Posner [12] analyzed the sea clutter data in X-band, and used Pareto distribution to fit data. They found that the fitting effect of the tail was better than *K* distribution.

The paper is organized as follows—Section 2 introduces the preliminary including some geometric structure of statistical manifolds and some concepts of Bayesian inference. In Section 3, we introduce the geometric approaches for Bayesian inference by using α-parallel connection, and propose a geometric loss function based on geodesic distance. In Section 4, we prove that Pareto two-parameter model does not have general α-parallel prior. Then we adopt Jeffreys prior to provide the explicit expressions of estimations in the sense of minimal mean geodesic distance. Furthermore, we come up with theorems under Al-Bayyati’s loss function to obtain a new class of Bayesian estimations. The bounds of certain expressions without closed forms are given. Besides, we show the existence of the best parameter in Al-Bayyati’s loss function. In Section 5, we show the advantages of the proposed Bayesian estimations and the posterior prediction distributions.

## 2. Preliminaries

### 2.1. *α*-Parallel Prior

**Definition** **1** ([7,13])**.** *For a statistical manifold M={p(x;θ)|∫p(x;θ)dx=1,p(x;θ)>0,$\theta \in \Theta \subset R^{d}\}$, define an affine connection $\nabla^{\left(\alpha \right)}$ on M with the following coefficients*

$$\begin{array}{c} \Gamma_{jk}^{(\alpha )i}:=\Gamma_{jk}^{(0)i}+\frac{1-\alpha }{2}T_{sjk}g^{is},\;\Gamma_{jk}^{(0)i}:=\Gamma_{s:jk}^{\left(0\right)}g^{is}, \end{array}$$

*where α∈R is an arbitrary real number, $g_{ij}=E\left[\partial_{i}l\partial_{j}l\right]$, $\left(g^{ij}\right)$ is the inverse matrix $\left(g_{ij}\right)$, $\Gamma_{s:jk}^{\left(0\right)}=E\left[\partial_{s}l\partial_{j}\partial_{k}l\right],T_{sjk}=E\left[\partial_{s}l\partial_{j}l\partial_{k}l\right]$, l:=logp(x;θ) denotes the log likelihood function and $E\left[\cdot \right]$ denotes the expectation with respect to the observation x.*

**Definition** **2** ([7])**.** *An affine connection* ∇ *is called locally equiaffine if around each point x of M, there is a parallel volume element, that is, a nonvanishing d-form w such that ∇w=0.*

*An equiaffine connection* ∇ *on M is a torsion-free affine connection with a parallel volume element w on M.*

*If w is a volume element on M such that ∇w=0, then we say that $\left(\nabla ,w\right)$ is an affine structure on M.*

For a statistical manifold *M*, we may represent the α-parallel volume element *w* as

$$\begin{array}{c} w=\pi \left(\theta \right)d\theta^{1}\wedge \cdot \cdot \cdot \wedge d\theta^{d} \end{array}$$

for a certain coordinate $\theta =\left(\theta^{1},\cdot \cdot \cdot ,\theta^{d}\right)\in \Theta \subset R^{d}$, where π is the volume form on the whole manifold. We take π(θ) as a prior distribution on the parameter space Θ.

**Definition** **3** ([7])**.** *In a statistically equiaffine manifold, for a fixed α∈R, we call the above form of π an α-parallel prior.*

When α=1, 1-parallel prior is called maximum likelihood estimation (MLE) prior proposed by J.A. Hartigan [5]. Note that there always exists a $\nabla^{\left(0\right)}$-parallel volume element $w\propto \sqrt{g(\theta )}d\theta^{1}\wedge \cdot \cdot \cdot \wedge d\theta^{d}$, where *g* is the determinant of the Fisher metric, the invariant volume element in a Riemannian manifold $\left(M,\left(g_{ij}\right)\right)$. This prior distribution $\pi \propto \sqrt{g(\theta )}$ is called the Jeffreys prior.

J. Takeuchi and S. Amari gave a sufficient and necessary condition for the existence of α-parallel prior.

**Proposition** **1** ([6])**.** *For a statistical manifold M with the α connection $\nabla^{\left(\alpha \right)}$, if α≠0, then there exists an α-parallel prior if and only if*

(1) $$\begin{array}{c} \partial_{i}T_{j}-\partial_{j}T_{i}=0 \end{array}$$

*where $T_{i}:=T_{ikl}g^{kl}$.*

### 2.2. Bayesian Inference

For the random variate x subject to the distribution p(x;θ), and let π(θ) be the prior distribution of θ. The posterior distribution π(θ|x) is given by the formula

$$\begin{array}{c} \pi (\theta |x)=\frac{p(x;\theta )\pi (\theta )}{\int_{\Theta }p\left(x;\theta \right)\pi \left(\theta \right)\;d\theta }. \end{array}$$

Now, we introduce some notations for later uses. Let ${\hat{\theta }}_{MD}$ be the maximum posterior estimation. Let ${\hat{\theta }}_{Me}$ be the posterior median estimation which is the median of the posterior distribution. Let ${\hat{\theta }}_{E}$ be the posterior expectation estimation which is the expectation of the posterior distribution.

These three estimations are also known as Bayesian estimations of θ. When $\hat{\theta }={\hat{\theta }}_{E}$, the posterior mean square value reaches the minimum. Hence ${\hat{\theta }}_{E}=E\left[\theta |x\right]$ is often taken as the Bayesian estimation.

Let the random variable X∼p(x;θ). If one does not know the observation data, the marginal distribution m(x) is also known as the prior prediction distribution. If one obtains the observation data $x=(x_{1},\cdot \cdot \cdot ,x_{n})$, the distribution of unknown observation values could be obtained by the posterior distribution π(θ|x):

1. Predict the future observations of the same population $p(\tilde{x};\theta )$
  $$\begin{array}{c} m(\tilde{x}|x)=\int_{\Theta }p\left(\tilde{x};\theta \right)\pi \left(\theta |x\right)\;d\theta . \end{array}$$
2. Predict the observations of another population g(z;θ)
  $$\begin{array}{c} m(z|x)=\int_{\Theta }g\left(z;\theta \right)\pi \left(\theta |x\right)\;d\theta , \end{array}$$
  where $m(\tilde{x}|x)$ or m(z|x) is called the posterior predictive distribution.

## 3. The Geometric Approaches for Bayesian Inference

In this section, we introduce the basic methods of Bayesian inference with geometric means. The idea of geometry is embodied in the selection of priors and loss functions.

### 3.1. The Geometric Prior

The idea of geometric methods is to extend the uniform distribution naturally and construct geometric priors suitable for multidimensional and measure infinite-dimensional parameter space according to the idea that probability measure is proportional to volume element. The studied probability distribution family can be regarded as a statistical manifold with Riemannian metrics.

Fisher information matrix is the most widely used Riemannian metric on statistical manifolds, and the prior generated by its corresponding volume element is Jeffreys prior. α-connection is a natural extension of the Levi-Civita connection corresponding to Fisher information matrix. Its corresponding volume element is α-parallel volume element, and the generated prior is called α-parallel prior. In particular, the 0-parallel prior is the Jeffreys prior.

α-parallel prior reflects the intrinsic property of the model and does not depend on the selection of parameters. Although Jeffreys prior must exist, general α-parallel prior does not necessarily exist. (1) gives the necessary and sufficient conditions for the existence of general α-parallel prior.

Therefore, when one deals with Bayesian inference by geometric methods, the first step is to select the appropriate geometric priors, that is, to verify the existence of α-parallel prior in a specific statistical manifold.

With Riemannian metric, we can acquire geometric information of the statistical manifold, such as connection, curvature, geodesic and geodesic distance [14,15]. Through geometric priors, the joint posterior density of the parameters can be obtained, and then the corresponding Bayesian estimation and Bayesian posterior prediction are carried out [16].

### 3.2. The Geometric Loss Functions

In this subsection, we show the geometric meaning of the common Bayesian estimations and propose a new geometric approach of choosing loss functions.

**Proposition** **2.** *Suppose that $\theta =\left(\theta_{1},\cdots ,\theta_{d}\right)\in \Theta \subset R^{d}$. Let π(θ|x) be the joint posterior distribution and $\hat{\theta }=\left({\hat{\theta }}_{1},\cdots ,{\hat{\theta }}_{d}\right)$ be the estimation of θ. For the loss function $l_{1}\left(\theta ,\hat{\theta }\right)=\sum_{i=1}^{d}\left|{\hat{\theta }}_{i}-\theta_{i}\right|$, the corresponding estimation is ${\hat{\theta }}_{Me}=\left({\hat{\theta }}_{1Me},\cdots ,{\hat{\theta }}_{dMe}\right)$. For the loss function $l_{2}\left(\theta ,\hat{\theta }\right)=\sum_{i=1}^{n}{\left({\hat{\theta }}_{i}-\theta_{i}\right)}^{2}$, the corresponding estimation is ${\hat{\theta }}_{E}=\left({\hat{\theta }}_{1E},\cdots ,{\hat{\theta }}_{dE}\right)$.*

**Proof.**  Denote $d\overset{ˇ}{\theta_{i}}=d\theta_{1}\cdots d\theta_{i-1}d\theta_{i+1}\cdots d\theta_{d}$. Let $\pi (\theta_{i}|x)$ be the marginal posterior density and $\mathrm{Pr}(\theta_{i}\leq t|x)$ be its cumulative distribution. Assume that $\mathrm{Pr}(\theta_{i}\leq a|x)=0$ and $\mathrm{Pr}(\theta_{i}\leq b|x)=1$, where a,b∈R and they may be infinite.

For the loss function $l_{1}\left(\theta ,\hat{\theta }\right)$, we define

$$\begin{array}{cc} R_{1}\left(\theta ,\hat{\theta }\right) & =\int_{\Theta }l_{1}\left(\theta ,\hat{\theta }\right)\pi \left(\theta |x\right)d\theta \\ & =\left(\int_{a}^{{\hat{\theta }}_{i}}-\int_{{\hat{\theta }}_{i}}^{b}\right)\int_{\overset{ˇ}{\theta_{i}}}\left({\hat{\theta }}_{i}-\theta_{i}\right)\pi \left(\theta |x\right)d\overset{ˇ}{\theta_{i}}d\theta_{i}+\sum_{j\neq i}\int_{\Theta }\left|{\hat{\theta }}_{j}-\theta_{j}\right|\pi \left(\theta |x\right)d\theta \\ & ={\hat{\theta }}_{i}\left(\int_{a}^{{\hat{\theta }}_{i}}-\int_{{\hat{\theta }}_{i}}^{b}\right)\pi \left(\theta_{i}|x\right)d\theta_{i}-\left(\int_{a}^{{\hat{\theta }}_{i}}-\int_{{\hat{\theta }}_{i}}^{b}\right)\theta_{i}\pi \left(\theta_{i}|x\right)d\theta_{i}+\sum_{j\neq i}\int_{\Theta }\left|{\hat{\theta }}_{j}-\theta_{j}\right|\pi \left(\theta |x\right)d\theta . \end{array}$$

Then we get

$$\begin{array}{c} \frac{\partial R_{1}}{\partial {\hat{\theta }}_{i}}=\int_{a}^{{\hat{\theta }}_{i}}\pi \left(\theta_{i}|x\right)d\theta_{i}-\int_{{\hat{\theta }}_{i}}^{b}\pi \left(\theta_{i}|x\right)d\theta_{i}. \end{array}$$

Let $\frac{\partial R_{1}}{\partial {\hat{\theta }}_{i}}=0$, we have ${\hat{\theta }}_{i}={\hat{\theta }}_{iMe}$.

For the loss function $l_{2}\left(\theta ,\hat{\theta }\right)$, we define

$$\begin{array}{cc} R_{2}\left(\theta ,\hat{\theta }\right)\; & =\int_{\Theta }l_{2}\left(\theta ,\hat{\theta }\right)\pi \left(\theta |x\right)d\theta =\sum_{i=1}^{d}\int_{\Theta }{\left({\hat{\theta }}_{i}-\theta_{i}\right)}^{2}\pi \left(\theta |x\right)d\theta . \end{array}$$

Then we obtain

$$\begin{array}{c} \frac{\partial R_{2}}{\partial {\hat{\theta }}_{i}}=2\int_{a}^{b}\left({\hat{\theta }}_{i}-\theta_{i}\right)\pi \left(\theta_{i}|x\right)d\theta_{i} \end{array}$$

Hence, $\frac{\partial R_{2}}{\partial {\hat{\theta }}_{i}}=0$ implies that ${\hat{\theta }}_{i}={\hat{\theta }}_{iE}$.  □

If the loss function is the distance induced by ${∥\cdot ∥}_{1}$, then by Proposition 2 we see that the corresponding risk function represents the average distance between the estimated value and the true value. Besides, the posterior median estimation of parameters minimizes risk function, which means that this estimation has the minimum mean distance from the posterior density.

If the loss function is the distance induced by ${∥\cdot ∥}_{2}$, then the corresponding risk function represents the average value of the square of the distance between the estimated value and the true value. The obtained estimation is the posterior expectation of parameters, which has the minimum mean square error from the posterior density.

These two kinds of loss functions above are distances or increasing functions of distances in $R^{n}$. However, in the parameter space endowed with corresponding Riemannian metric, the distance between two points is geodesic distance instead of Euclidean distance.

Hence, in order to make the estimation more accurate, we take the geodesic distance or its increasing function as a loss function, the corresponding risk function represents the geodesic distance between the estimated value and the true value. Before that, we need the following definition.

**Definition** **4.** *(Mean Geodesic Estimation) Assume that the statistical manifold M={p(x;θ)} is equipped with Fisher Riemannian metric $\left(g_{ij}\right)$. Let π(θ|x) be the joint posterior distribution and $d(\theta ,\hat{\theta })$ be the geodesic distance between θ and $\hat{\theta }$, where $\hat{\theta }$ is the estimation of θ. Let F: R→R be an increasing function. Denote $D\left(\theta ,\hat{\theta }\right)=F\circ d\left(\theta ,\hat{\theta }\right)$. The risk function with the loss function $D(\theta ,\hat{\theta })$ is*

(2) $$R\left(\theta ,\hat{\theta }\right)=\int_{\Theta }D\left(\theta ,\hat{\theta }\right)\pi \left(\theta |x\right)d\theta .$$

*The estimation minimizing $R(\theta ,\hat{\theta })$ is called mean geodesic estimation (MGE) and denoted by ${\hat{\theta }}_{MGE}$.*

The geometric priors, the corresponding geodesic distance and the corresponding Bayesian inference depend on the choice of the Riemannian metric. Hence choosing a proper Riemannian metric is of great importance to Bayesian inference.

## 4. Bayesian Inference on Pareto Model

### 4.1. The Geometric Structure of Pareto Two-Parameter Model

The probability density function of Pareto two-parameter distribution satisfies

(3) $$\begin{array}{c} p\left(x;\alpha ,\beta \right)=\frac{\beta \alpha^{\beta }}{x^{\beta +1}}I_{\left[x\geq \alpha \right]},\;\alpha >0,\beta >0, \end{array}$$

where α is called the scale parameter and β is called the shape parameter.

Its logarithmic likelihood function is

(4) $$\begin{array}{c} l(x;\alpha ,\beta )=\log p(x;\alpha ,\beta )=\log\beta +\beta \log\alpha -(\beta +1)\log x \end{array}$$

Noting that the Pareto distribution family does not meet the common regularity condition, hence the Fisher-Rao metric on the Pareto distribution family is not equal to the negative Hessian matrix.

Furthermore, from References [17,18] we can get the geometric structure of Pareto model. On Pareto two-parameter distribution family, the tensor form of Fisher-Rao metric satisfies

(5) $$\begin{array}{c} g=\frac{\beta^{2}}{\alpha^{2}}d\alpha ⊗d\alpha +\frac{1}{\beta^{2}}d\beta ⊗d\beta , \end{array}$$

which is isometric to the upper half of the Poincar$\overset{´}{e}$ plane. Hence, Pareto two-parameter model (P,g) is a Riemannian manifold endowed with Riemannian metric *g*. The volume form, the connection form, the curvature form, the Christoffel symbols and the geodesic distance formula on (P,g) are given as follows

(6) $$\begin{array}{c} dv=\theta^{1}\wedge \theta^{2}=\frac{1}{\alpha }d\alpha \wedge d\alpha \end{array}$$

(7) $$\begin{array}{c} w_{2}^{1}=\frac{\beta }{\alpha }d\alpha \end{array}$$

(8) $$\begin{array}{c} \Omega_{2}^{1}=dw_{2}^{1}=-\frac{1}{\alpha }d\alpha \wedge d\alpha =-Kdv \end{array}$$

(9) $$\begin{array}{c} \left\{\begin{array}{c} \nabla_{\partial_{1}}\partial_{1}=-\frac{1}{\alpha }\partial_{1}-\frac{\beta^{3}}{\alpha^{2}}\partial_{2}\\ \nabla_{\partial_{2}}\partial_{1}=\nabla_{\partial_{1}}\partial_{2}=\frac{1}{\beta }\partial_{1}\\ \nabla_{\partial_{2}}\partial_{2}=-\frac{1}{\beta }\partial_{2} \end{array}\right. \end{array}$$

(10) $$\begin{array}{c} d\left(\left(\alpha_{0},\beta_{0}\right),\left(\alpha_{1},\beta_{1}\right)\right)=\mathrm{arcosh}\left(1+\frac{\beta_{0}\beta_{1}{\left(\log\alpha_{0}-\log\alpha_{1}\right)}^{2}}{2}+\frac{{\left(\beta_{0}-\beta_{1}\right)}^{2}}{2\beta_{0}\beta_{1}}\right). \end{array}$$

### 4.2. The Existence of *α*-Parallel Prior on Pareto Two-Parameter Model

**Theorem** **1.** *When α≠0, Pareto two-parameter model does not have any α-parallel prior.*

**Proof.**  Denote $\partial_{1}=\frac{\partial }{\partial \alpha },\;\partial_{2}=\frac{\partial }{\partial \beta }$. Then by calculation, we can obtain

(11) $$\begin{array}{c} \left\{\begin{array}{c} T_{111}=E\left[\partial_{1}l\partial_{1}l\partial_{1}l\right]=\frac{\beta^{3}}{\alpha^{3}}\\ T_{222}=E\left[\partial_{2}l\partial_{2}l\partial_{2}l\right]=-\frac{2}{\beta^{3}}\\ T_{112}=T_{121}=T_{211}=E\left[\partial_{1}l\partial_{1}l\partial_{2}l\right]=0\\ T_{122}=T_{221}=T_{212}=E\left[\partial_{1}l\partial_{2}l\partial_{2}l\right]=\frac{1}{\alpha \beta } \end{array}\right. \end{array}$$

Hence, we get

$$\begin{array}{c} \left\{\begin{array}{c} T_{1}=T_{1ik}g^{ik}=\frac{2\beta }{\alpha }\\ T_{2}=T_{2ik}g^{ik}=-\frac{2}{\beta } \end{array}\right. \end{array}$$

and

$$\begin{array}{cc} \partial_{i}T_{j}-\partial_{j}T_{i} & =\pm \frac{2}{\alpha }d\beta \wedge d\alpha . \end{array}$$

It is obvious that α≠0 means $\partial_{i}T_{j}-\partial_{j}T_{i}\neq 0$. Therefore, according to Proposition 1, we find that Pareto two-parameter model does not have any α-parallel prior when α≠0.  □

From Theorem 1, we see that Pareto two-parameter model only has the 0-parallel prior (Jeffreys prior). Its Jeffreys prior π(α,β) satisfies $\pi \left(\alpha ,\beta \right)\propto \sqrt{\frac{\alpha^{2}}{\beta^{2}}\frac{1}{\beta^{2}}}=\frac{1}{\alpha },\alpha >0,\beta >0$, which is a generalized prior density whose integral is infinite. We assume that $\pi \left(\alpha ,\beta \right)=\frac{1}{\alpha },$α>0,β>0.

### 4.3. Bayesian Estimations of Pareto Model

Before we proceed, we state necessary results from Reference [17].

The joint probability density of a simple random sample on Pareto model is

(12) $$\begin{array}{c} p\left(x;\alpha ,\beta \right)=\beta^{n}\alpha^{n\beta }{\left(\prod_{i=1}^{n}x_{i}\right)}^{-\beta -1}I_{\left[\min_{i=1}^{n}x_{i}\geq \alpha \right]}. \end{array}$$

The posterior distribution of Pareto model under Jeffreys prior is obtained by Bayesian formula

(13) $$\begin{array}{c} \pi \left(\alpha ,\beta |x\right)=\frac{n{\left(q_{2}\left(x\right)-n\log q_{1}\left(x\right)\right)}^{n}}{\tau (n)}\beta^{n}\alpha^{n\beta -1}\exp\left(-q_{2}\left(x\right)\beta \right)I_{\left[0\leq \alpha \leq q_{1}\left(x\right)\right]}, \end{array}$$

where $q_{1}\left(x\right)=\min_{i=1}^{n}x_{i},\;q_{2}\left(x\right)=\sum_{i=1}^{n}\log x_{i}$. Furthermore, by calculation we can see that the maximum likelihood estimation and the maximum posterior estimation of α,β are given as

(14) $$\begin{array}{c} \left\{\begin{array}{c} {\hat{a}}_{MLE}={\hat{a}}_{MD}=q_{1}\left(x\right)\\ {\hat{\beta }}_{MLE}={\hat{\beta }}_{MD}=\frac{n}{\sum_{i=1}^{n}\log x_{i}-n\log\left(\min_{i=1}^{n}x_{i}\right)}=\frac{n}{q_{2}\left(x\right)-n\log q_{1}\left(x\right)}. \end{array}\right. \end{array}$$

The marginal posterior density of α determined by the joint posterior density π(α,β|x) is

(15) $$\begin{array}{c} \pi \left(\alpha |x\right)=\frac{n^{2}{\left(q_{2}\left(x\right)-n\log q_{1}\left(x\right)\right)}^{n}}{\alpha {\left(q_{2}\left(x\right)-n\log\alpha \right)}^{n+1}}I_{\left[0\leq \alpha \leq q_{1}\left(x\right)\right]} \end{array}$$

and its cumulative distribution function is

(16) $$\begin{array}{c} \mathrm{Pr}\left(\alpha \leq t|x\right)={\left(1+{\hat{\beta }}_{MLE}\log\frac{{\hat{\alpha }}_{MLE}}{t}\right)}^{-n},\;0\leq t\leq q_{1}\left(x\right). \end{array}$$

The marginal posterior density of β determined by the joint posterior density π(α,β|x) is

(17) $$\begin{array}{c} \pi \left(\beta |x\right)=\frac{\beta^{n-1}{\left(q_{2}\left(x\right)-n\log q_{1}\left(x\right)\right)}^{n}}{\tau (n)}\exp\left(-\left(q_{2}\left(x\right)-n\log q_{1}\left(x\right)\right)\beta \right). \end{array}$$

Under posterior distribution (13), when β is known, the conditional posterior density of α is

(18) $$\begin{array}{c} \pi \left(\alpha |x,\beta \right)=\frac{n\beta \alpha^{n\beta -1}}{q_{1}^{n\beta }\left(x\right)}I_{\left[0\leq \alpha \leq q_{1}\left(x\right)\right]} \end{array}$$

and its cumulative distribution function is

(19) $$\begin{array}{c} \mathrm{Pr}\left(\alpha \leq t|x,\beta \right)={\left(\frac{t}{q_{1}\left(x\right)}\right)}^{n\beta },\;0\leq t\leq q_{1}\left(x\right). \end{array}$$

When α is known, the conditional posterior density of β is

(20) $$\begin{array}{c} \pi \left(\beta |x,\alpha \right)=\frac{\beta^{n}{\left(q_{2}\left(x\right)-n\log\alpha \right)}^{n+1}}{\tau (n+1)}\exp\left(-(q_{2}\left(x\right)-n\log\alpha )\beta \right). \end{array}$$

#### 4.3.1. Mean Geodesic Estimation

The geodesic distance between (α,β) and $\left(\hat{\alpha },\hat{\beta }\right)$ is expressed as

(21) $$\begin{array}{c} d\left(\left(\alpha ,\beta \right),\left(\hat{\alpha },\hat{\beta }\right)\right)=\mathrm{arcosh}\left(1+\frac{\beta \hat{\beta }{\left(\log\alpha -\log\hat{\alpha }\right)}^{2}}{2}+\frac{{\left(\beta -\hat{\beta }\right)}^{2}}{2\beta \hat{\beta }}\right). \end{array}$$

Hence, the distance function is a monotone function of

(22) $$1+\frac{\beta \hat{\beta }{\left(\log\alpha -\log\hat{\alpha }\right)}^{2}}{2}+\frac{{\left(\beta -\hat{\beta }\right)}^{2}}{2\beta \hat{\beta }}.$$

We denote (22) as

(23) $$\begin{array}{c} D\left(\left(\alpha ,\beta \right),\left(\hat{\alpha },\hat{\beta }\right)\right)=1+\frac{\beta \hat{\beta }{\left(\log\alpha -\log\hat{\alpha }\right)}^{2}}{2}+\frac{{\left(\beta -\hat{\beta }\right)}^{2}}{2\beta \hat{\beta }}. \end{array}$$

By the discussion in the Section 3, when α and β are unknown, $D(\left(\alpha ,\beta \right),\left(\hat{\alpha },\hat{\beta }\right))$ can be taken as the loss funtion, and the estimations ${\hat{\alpha }}_{MGE}$ and ${\hat{\beta }}_{MGE}$ in the sense of minimum mean geodesic distance can be obtained. When $\beta =\beta_{0}$ or $\alpha =\alpha_{0}$, $D_{\beta_{0}}\left(\left(\alpha ,\beta_{0}\right),\left(\hat{\alpha },\beta_{0}\right)\right)$ or $D_{\alpha_{0}}\left(\left(\alpha_{0},\beta \right),\left(\alpha_{0},\hat{\beta }\right)\right)$ can be taken as the loss function respectively, and we can get the mean geodesic estimation ${\hat{\alpha }}_{MGE}\left(x|\beta \right)$ or ${\hat{\beta }}_{MGE}\left(x|\alpha \right)$.

**Theorem** **2.** *When α and β are unknown, we have*

$$\begin{array}{c} {\hat{\alpha }}_{MGE}=\frac{{\hat{\alpha }}_{MLE}}{\exp\left(\frac{1}{n{\hat{\beta }}_{MLE}}\right)},\;{\hat{\beta }}_{MGE}=\sqrt{\frac{2n^{2}\left(n-1\right)}{2n^{3}+n+1}}{\hat{\beta }}_{MLE}. \end{array}$$

**Proof.**  Denote $f\left(\alpha \right)=E_{\beta }\left[\pi \left(\alpha ,\beta |x\right)\right]$, then have

$$\begin{array}{c} f\left(\alpha \right)=\frac{n^{2}\left(n+1\right){\left(q_{2}\left(x\right)-n\log q_{1}\left(x\right)\right)}^{n}}{\alpha {\left(q(x)-n\log\alpha \right)}^{n+2}},\;0\leq \alpha \leq q_{1}\left(x\right). \end{array}$$

Let $F\left(t\right)=\int_{0}^{t}f\left(\alpha \right)d\alpha$, then we have

$$\begin{array}{c} F\left(t\right)=\frac{n{\left(q_{2}\left(x\right)-n\log q_{1}\left(x\right)\right)}^{n}}{{\left(q(x)-n\log t\right)}^{n+1}},\;0\leq t\leq q_{1}\left(x\right). \end{array}$$

Denote $g\left(t\right)=\frac{F(t)}{t}$, then

$$\begin{array}{c} G\left(s\right)=\int_{0}^{s}g\left(t\right)\mathrm{dt}=\frac{{\left(q_{2}\left(x\right)-n\log q_{1}\left(x\right)\right)}^{n}}{n{\left(q(x)-n\log s\right)}^{n}},\;0\leq s\leq q_{1}\left(x\right). \end{array}$$

Denote $h\left(t\right)=\frac{G(t)}{t}$, then have

$$\begin{array}{c} H\left(v\right)=\int_{0}^{v}h\left(t\right)\mathrm{dt}=\frac{{\left(q_{2}\left(x\right)-n\log q_{1}\left(x\right)\right)}^{n}}{n^{2}\left(n-1\right){\left(q(x)-n\log v\right)}^{n-1}},\;0\leq v\leq q_{1}\left(x\right). \end{array}$$

The risk function is

$$\begin{array}{c} R\left(\left(\alpha ,\beta \right),\left(\hat{\alpha },\hat{\beta }\right)\right)=\int_{0}^{{\hat{\alpha }}_{MLE}}\int_{0}^{+\infty }D\left(\left(\alpha ,\beta \right),\left(\hat{\alpha },\hat{\beta }\right)\right)\pi \left(\alpha ,\beta |x\right)d\beta d\alpha . \end{array}$$

Firstly, we show that ${\hat{\alpha }}_{MGE}=\frac{{\hat{\alpha }}_{MLE}}{\exp\left(\frac{1}{n{\hat{\beta }}_{MLE}}\right)}$. Since

$$\begin{array}{c} \frac{\partial R}{\partial \hat{\alpha }}=\frac{\hat{\beta }}{\hat{\alpha }}\int_{0}^{{\hat{\alpha }}_{MLE}}\int_{0}^{+\infty }\left(\log\alpha -\log\hat{\alpha }\right)\beta \pi \left(\alpha ,\beta |x\right)d\beta d\alpha , \end{array}$$

when $\frac{\partial R}{\partial \hat{\alpha }}=0$, we have

$$\begin{array}{c} \log{\hat{\alpha }}_{MGE}=\frac{\int_{0}^{{\hat{\alpha }}_{MLE}}\log\alpha \cdot E_{\beta }\left[\pi \left(\alpha ,\beta |x\right)\right]d\alpha }{\int_{0}^{{\hat{\alpha }}_{MLE}}E_{\beta }\left[\pi \left(\alpha ,\beta |x\right)\right]d\alpha }. \end{array}$$

Combining

$$\begin{array}{c} \int_{0}^{{\hat{\alpha }}_{MLE}}E_{\beta }\left[\pi \left(\alpha ,\beta |x\right)\right]d\alpha =F\left({\hat{\alpha }}_{MLE}\right)={\hat{\beta }}_{MLE} \end{array}$$

with

$$\begin{array}{cc} \int_{0}^{{\hat{\alpha }}_{MLE}}\log\alpha \cdot E_{\beta }\left[\pi \left(\alpha ,\beta |x\right)\right]d\alpha & =\int_{0}^{{\hat{\alpha }}_{MLE}}\log tdF\left(t\right)\\ & ={\hat{\beta }}_{MLE}\log{\hat{\alpha }}_{MLE}-G\left({\hat{\alpha }}_{MLE}\right)\\ & ={\hat{\beta }}_{MLE}\left(\log{\hat{\alpha }}_{MLE}-\frac{1}{n{\hat{\beta }}_{MLE}}\right) \end{array}$$

we get

$$\begin{array}{c} {\hat{\alpha }}_{MGE}=\frac{{\hat{\alpha }}_{MLE}}{\exp\left(\frac{1}{n{\hat{\beta }}_{MLE}}\right)}. \end{array}$$

Secondly, we will show that ${\hat{\beta }}_{MGE}=\sqrt{\frac{2n^{2}\left(n-1\right)}{2n^{3}+n+1}}{\hat{\beta }}_{MLE}$. In fact, from

$$\begin{array}{c} \frac{\partial R}{\partial \hat{\beta }}=\int_{0}^{{\hat{\alpha }}_{MLE}}\int_{0}^{+\infty }\left(\frac{\beta {\left(\log\alpha -\log\hat{\alpha }\right)}^{2}}{2}-\frac{\beta }{{\hat{\beta }}^{2}}+\frac{1}{\beta }\right)\pi \left(\alpha ,\beta |x\right)d\beta d\alpha \end{array}$$

we see that when $\frac{\partial R}{\partial \hat{\beta }}=0$, we have

$$\begin{array}{cc} \frac{{\hat{\beta }}_{MLE}}{{\hat{\beta }}_{MGE}^{2}} & =\frac{F({\hat{\alpha }}_{MLE})}{{\hat{\beta }}_{MGE}^{2}}=\int_{0}^{{\hat{\alpha }}_{MLE}}\int_{0}^{+\infty }\frac{1}{\beta }\pi \left(\alpha ,\beta |x\right)d\beta d\alpha +\frac{1}{2}\int_{0}^{{\hat{\alpha }}_{MLE}}{\left(\log\alpha -\log\hat{\alpha }\right)}^{2}f\left(\alpha \right)d\alpha . \end{array}$$

Noting that

$$\begin{array}{cc} \int_{0}^{{\hat{\alpha }}_{MLE}}\int_{0}^{+\infty }\frac{1}{\beta }\pi \left(\alpha ,\beta |x\right)d\beta d\alpha & =\int_{0}^{+\infty }\frac{1}{\beta }\pi \left(\beta |x\right)d\beta \\ \; & =\frac{{\left(q_{2}\left(x\right)-n\log q_{1}\left(x\right)\right)}^{n}}{\tau (n)}\int_{0}^{+\infty }\beta^{n-2}\exp\left(-(q_{2}\left(x\right)-n\log q_{1}\left(x\right))\beta \right)d\beta \\ \; & =\frac{q_{2}\left(x\right)-n\log q_{1}\left(x\right)}{n-1}\\ \; & =\frac{n}{\left(n-1\right){\hat{\beta }}_{MLE}} \end{array}$$

and

$$\begin{array}{cc} \int_{0}^{{\hat{\alpha }}_{MLE}}{\left(\log\alpha -\log\hat{\alpha }\right)}^{2}f\left(\alpha \right)d\alpha & =\int_{0}^{{\hat{\alpha }}_{MLE}}{\left(\log t-\log\hat{\alpha }\right)}^{2}\mathrm{dF}(t)\\ \; & ={\hat{\beta }}_{MLE}{\left(\log{\hat{\alpha }}_{MLE}-\log\hat{\alpha }\right)}^{2}-2\int_{0}^{{\hat{\alpha }}_{MLE}}\left(\log t-\log\hat{\alpha }\right)g\left(t\right)\mathrm{dt}\\ \; & ={\hat{\beta }}_{MLE}\frac{1}{{\left(n{\hat{\beta }}_{MLE}\right)}^{2}}-2G\left({\hat{\alpha }}_{MLE}\right)\frac{1}{n{\hat{\beta }}_{MLE}}+2H\left({\hat{\alpha }}_{MLE}\right)\\ \; & =-\frac{1}{n^{2}{\hat{\beta }}_{MLE}}+\frac{2}{n\left(n-1\right){\hat{\beta }}_{MLE}}\\ \; & =\frac{n+1}{n^{2}\left(n-1\right){\hat{\beta }}_{MLE}} \end{array}$$

we have

$$\begin{array}{c} \frac{{\hat{\beta }}_{MLE}}{{\hat{\beta }}_{MGE}^{2}}=\frac{n}{\left(n-1\right){\hat{\beta }}_{MLE}}+\frac{n+1}{2n^{2}\left(n-1\right){\hat{\beta }}_{MLE}}=\frac{2n^{3}+n+1}{2n^{2}\left(n-1\right){\hat{\beta }}_{MLE}}. \end{array}$$

As a result, we obtain ${\hat{\beta }}_{MGE}=\sqrt{\frac{2n^{2}\left(n-1\right)}{2n^{3}+n+1}}{\hat{\beta }}_{MLE}$.  □

**Theorem** **3.** *When β is known, we have ${\hat{\alpha }}_{MGE}\left(x|\beta \right)=\frac{{\hat{\alpha }}_{MLE}}{\exp\left(\frac{1}{n\beta }\right)}$. And when α is known, we have ${\hat{\beta }}_{MGE}\left(x|\alpha \right)=\frac{\sqrt{n(n+1)}}{q_{2}\left(x\right)-n\log\alpha }$.*

**Proof.**  When β is known, the risk function is

$$\begin{array}{c} R\left(\left(\alpha ,\beta \right),\left(\hat{\alpha },\beta \right)\right)=\int_{0}^{{\hat{\alpha }}_{MLE}}D_{\beta }\left(\left(\alpha ,\beta \right),\left(\hat{\alpha },\beta \right)\right)\pi \left(\alpha |x,\beta \right)d\alpha . \end{array}$$

Since

$$\begin{array}{c} \frac{dR}{d\hat{\alpha }}=\frac{\beta^{2}}{\hat{\alpha }}\int_{0}^{{\hat{\alpha }}_{MLE}}\left(\log\alpha -\log\hat{\alpha }\right)\pi \left(\alpha |x,\beta \right)d\alpha \end{array}$$

when $\frac{dR}{d\hat{\alpha }}=0$, we have

$$\begin{array}{cc} \log{\hat{\alpha }}_{MGE}\left(x|\beta \right) & =\int_{0}^{{\hat{\alpha }}_{MLE}}\log\alpha \cdot \pi \left(\alpha |x,\beta \right)d\alpha \\ \; & =\int_{0}^{{\hat{\alpha }}_{MLE}}\log t\mathrm{dPr}\left(\alpha \leq t|x,\beta \right)\\ \; & =\log{\hat{\alpha }}_{MLE}-\int_{0}^{{\hat{\alpha }}_{MLE}}\frac{1}{t}\mathrm{Pr}\left(\alpha \leq t|x,\beta \right)dt\\ \; & =\log{\hat{\alpha }}_{MLE}-\frac{1}{n\beta }. \end{array}$$

Hence

$$\begin{array}{c} {\hat{\alpha }}_{MGE}\left(x|\beta \right)=\frac{{\hat{\alpha }}_{MLE}}{\exp\left(\frac{1}{n\beta }\right)}. \end{array}$$

When *α* is known, the risk function is

$$\begin{array}{c} R\left(\left(\alpha ,\beta \right),\left(\alpha ,\hat{\beta }\right)\right)=\int_{0}^{+\infty }D_{\alpha }\left(\left(\alpha ,\beta \right),\left(\alpha ,\hat{\beta }\right)\right)\pi \left(\beta |x,\alpha \right)d\beta . \end{array}$$

Since

$$\begin{array}{c} \frac{dR}{d\hat{\beta }}=\int_{0}^{+\infty }\left(-\frac{\beta }{{\hat{\beta }}^{2}}+\frac{1}{\beta }\right)\pi \left(\beta |x,\alpha \right)d\beta , \end{array}$$

when $\frac{dR}{d\hat{\beta }}=0$, we have

$$\begin{array}{c} {\hat{\beta }}_{MGE}\left(x|\alpha \right)=\frac{\int_{0}^{+\infty }\beta \pi \left(\beta |x,\alpha \right)d\beta }{\int_{0}^{+\infty }\frac{1}{\beta }\pi \left(\beta |x,\alpha \right)d\beta }. \end{array}$$

Noting that

$$\begin{array}{cc} \int_{0}^{+\infty }\beta \pi \left(\beta |x,\alpha \right)d\beta & =\frac{{\left(q_{2}\left(x\right)-n\log\alpha \right)}^{n+1}}{\tau (n+1)}\int_{0}^{+\infty }\beta^{n+1}\exp\left(-(q_{2}\left(x\right)-n\log\alpha )\beta \right)d\beta \\ \; & =\frac{n(n+1)}{{\left(q_{2}\left(x\right)-n\log\alpha \right)}^{2}}\frac{{\left(q_{2}\left(x\right)-n\log\alpha \right)}^{n+1}}{\tau (n+1)}\int_{0}^{+\infty }\beta^{n-1}\exp\left(-(q_{2}\left(x\right)-n\log\alpha )\beta \right)d\beta \\ \; & =\frac{n(n+1)}{{\left(q_{2}\left(x\right)-n\log\alpha \right)}^{2}}\int_{0}^{+\infty }\frac{1}{\beta }\pi \left(\beta |x,\alpha \right)d\beta \end{array}$$

hence we have ${\hat{\beta }}_{MGE}\left(x|\alpha \right)=\frac{\sqrt{n(n+1)}}{q_{2}\left(x\right)-n\log\alpha }$.  □

#### 4.3.2. Bayesian Estimations under Al-Bayyati’s Loss Function

The Al-Bayyati’s loss function was stated by Reference [19] as

(24) $$l\left(\hat{\theta },\theta \right)=\theta^{c}{\left(\hat{\theta }-\theta \right)}^{2},$$

where *c* is a real number. Next, we use Al-Bayyati’s loss function to derive the Bayesian estimation of Pareto model.

**Proposition** **3.** *Assume that θ∈Θ⊂R. Under Al-Bayyati’s loss function, the Bayesian estimation of parameter θ is given by*

(25) $$\begin{array}{c} {\hat{\theta }}_{B_{c}}=\frac{\int_{\Theta }\theta^{c+1}\pi \left(\theta |x\right)d\theta }{\int_{\Theta }\theta^{c}\pi \left(\theta |x\right)d\theta }. \end{array}$$

**Proof.**  Since the risk function

$$\begin{array}{c} R\left(\hat{\theta },\theta \right)=\int_{\Theta }\theta^{c}{\left(\hat{\theta }-\theta \right)}^{2}\pi \left(\theta |x\right)d\theta , \end{array}$$

we have

$$\begin{array}{c} \frac{dR(\hat{\theta },\theta )}{d\hat{\theta }}=\hat{\theta }\int_{\Theta }\theta^{c}\pi \left(\theta |x\right)d\theta -\int_{\Theta }\theta^{c+1}\pi \left(\theta |x\right)d\theta . \end{array}$$

Then we have

$$\begin{array}{c} {\hat{\theta }}_{B_{c}}=\frac{\int_{\Theta }\theta^{c+1}\pi \left(\theta |x\right)d\theta }{\int_{\Theta }\theta^{c}\pi \left(\theta |x\right)d\theta }, \end{array}$$

by letting $\frac{dR(\hat{\theta },\theta )}{d\hat{\theta }}=0$.  □

Using Al-Bayyati’s loss function, ${\hat{\alpha }}_{B_{c}}$ lacks the simple display expression. Thus we give the upper and lower bound estimations.

**Theorem** **4.** *Using Al-Bayyati’s loss function and assuming c≥0, we find that when β is unknown, then ${\hat{\alpha }}_{B_{c}}$ satisfies*

$$\begin{array}{c} \frac{\left(n{\hat{\beta }}_{MLE}+c\right)\left(\left(n-1\right){\hat{\beta }}_{MLE}-\left(c+1\right)\right)}{\left(n-1\right)n{\hat{\beta }}_{MLE}^{2}}{\hat{\alpha }}_{MLE}\leq {\hat{\alpha }}_{B_{c}}\leq \frac{\left(n-1\right)n{\hat{\beta }}_{MLE}^{2}}{\left(n{\hat{\beta }}_{MLE}+c+1\right)\left(\left(n-1\right){\hat{\beta }}_{MLE}-c\right)}{\hat{\alpha }}_{MLE}. \end{array}$$

*And when α is unknown, then*

$$\begin{array}{c} {\hat{\beta }}_{B_{c}}=\left(1+\frac{c}{n}\right){\hat{\beta }}_{MLE}. \end{array}$$

*Furthermore, there exists $c_{0}$ such that ${\hat{\beta }}_{B_{c_{0}}}=\beta_{0}$, where $\beta_{0}$ is the real value of shape parameter β.*

**Proof.**  When β is unknown, we have

$$\begin{array}{cc} \int_{0}^{{\hat{\alpha }}_{MLE}}\alpha^{c+1}\pi \left(\alpha |x\right)d\alpha & =\int_{0}^{{\hat{\alpha }}_{MLE}}t^{c+1}\mathrm{dPr}\left(\alpha \leq t|x\right)\\ \; & ={\left.\left(t^{c+1}\mathrm{Pr}\left(\alpha \leq t|x\right)\right)\right|}_{0}^{{\hat{\alpha }}_{MLE}}-\left(c+1\right)\int_{0}^{{\hat{\alpha }}_{MLE}}t^{c}\mathrm{Pr}\left(\alpha \leq t|x\right)dt\\ \; & ={\hat{\alpha }}_{MLE}^{c+1}-\left(c+1\right)\int_{0}^{{\hat{\alpha }}_{MLE}}t^{c}\mathrm{Pr}\left(\alpha \leq t|x\right)dt. \end{array}$$

Noting that

$$\begin{array}{cc} \int_{0}^{{\hat{\alpha }}_{MLE}}t^{c}\mathrm{dPr}\left(\alpha \leq t|x\right) & =\int_{0}^{{\hat{\alpha }}_{MLE}}t^{c}{\left(1+{\hat{\beta }}_{MLE}\log\frac{{\hat{\alpha }}_{MLE}}{t}\right)}^{-n}dt\\ \; & ={\hat{\alpha }}_{MLE}^{c+1}\int_{0}^{+\infty }\exp\left(-(c+1)u\right){\left(1+u{\hat{\beta }}_{MLE}\right)}^{-n}du\\ \; & \leq {\hat{\alpha }}_{MLE}^{c+1}\int_{0}^{+\infty }{\left(1+u{\hat{\beta }}_{MLE}\right)}^{-n}du\\ \; & =\frac{{\hat{\alpha }}_{MLE}^{c+1}}{\left(n-1\right){\hat{\beta }}_{MLE}} \end{array}$$

and that

$$\begin{array}{cc} \int_{0}^{{\hat{\alpha }}_{MLE}}t^{c}\mathrm{dPr}\left(\alpha \leq t|x\right) & ={\hat{\alpha }}_{MLE}^{c+1}\int_{0}^{+\infty }\exp\left(-(c+1)u\right){\left(1+u{\hat{\beta }}_{MLE}\right)}^{-n}du\\ \; & \geq {\hat{\alpha }}_{MLE}^{c+1}\int_{0}^{+\infty }\exp\left(-(c+1)u\right){\left(\exp\left(u{\hat{\beta }}_{MLE}\right)\right)}^{-n}du\\ \; & ={\hat{\alpha }}_{MLE}^{c+1}\int_{0}^{+\infty }\exp\left(-\left(n{\hat{\beta }}_{MLE}+c+1\right)u\right)du\\ \; & =\frac{{\hat{\alpha }}_{MLE}^{c+1}}{n{\hat{\beta }}_{MLE}+c+1} \end{array}$$

we have

$$\begin{array}{cc} \int_{0}^{{\hat{\alpha }}_{MLE}}\alpha^{c+1}\pi \left(\alpha |x\right)d\alpha & \geq {\hat{\alpha }}_{MLE}^{c+1}-\left(c+1\right)\frac{{\hat{\alpha }}_{MLE}^{c+1}}{\left(n-1\right){\hat{\beta }}_{MLE}}=\frac{\left(n-1\right){\hat{\beta }}_{MLE}-\left(c+1\right)}{\left(n-1\right){\hat{\beta }}_{MLE}}{\hat{\alpha }}_{MLE}^{c+1}\\ \int_{0}^{{\hat{\alpha }}_{MLE}}\alpha^{c+1}\pi \left(\alpha |x\right)d\alpha & \leq {\hat{\alpha }}_{MLE}^{c+1}-\frac{\left(c+1\right){\hat{\alpha }}_{MLE}^{c+1}}{n{\hat{\beta }}_{MLE}+c+1}=\frac{n{\hat{\beta }}_{MLE}}{n{\hat{\beta }}_{MLE}+c+1}{\hat{\alpha }}_{MLE}^{c+1}. \end{array}$$

Similarly, we can get

$$\begin{array}{c} \frac{\left(n-1\right){\hat{\beta }}_{MLE}-c}{\left(n-1\right){\hat{\beta }}_{MLE}}{\hat{\alpha }}_{MLE}^{c}\leq \int_{0}^{{\hat{\alpha }}_{MLE}}\alpha^{c}\pi \left(\alpha |x\right)d\alpha \leq \frac{n{\hat{\beta }}_{MLE}}{n{\hat{\beta }}_{MLE}+c}{\hat{\alpha }}_{MLE}^{c}. \end{array}$$

Furthermore, by Proposition 3, we have

$$\begin{array}{c} {\hat{\alpha }}_{B_{c}}=\frac{1-\left(c+1\right)\int_{0}^{+\infty }\exp\left(-(c+1)u\right){\left(1+u{\hat{\beta }}_{MLE}\right)}^{-n}du}{1-c\int_{0}^{+\infty }\exp\left(-cu\right){\left(1+u{\hat{\beta }}_{MLE}\right)}^{-n}du}{\hat{\alpha }}_{MLE} \end{array}$$

and

$$\begin{array}{cc} {\hat{\alpha }}_{B_{c}} & \geq \frac{\left(n-1\right){\hat{\beta }}_{MLE}-\left(c+1\right)}{\left(n-1\right){\hat{\beta }}_{MLE}}{\hat{\alpha }}_{MLE}^{c+1}\frac{n{\hat{\beta }}_{MLE}+c}{n{\hat{\beta }}_{MLE}}{\hat{\alpha }}_{MLE}^{-c}=\frac{\left(n{\hat{\beta }}_{MLE}+c\right)\left(\left(n-1\right){\hat{\beta }}_{MLE}-\left(c+1\right)\right)}{\left(n-1\right)n{\hat{\beta }}_{MLE}^{2}}{\hat{\alpha }}_{MLE}\\ {\hat{\alpha }}_{B_{c}} & \leq \frac{n{\hat{\beta }}_{MLE}}{n{\hat{\beta }}_{MLE}+c+1}{\hat{\alpha }}_{MLE}^{c+1}\frac{\left(n-1\right){\hat{\beta }}_{MLE}}{\left(n-1\right){\hat{\beta }}_{MLE}-c}{\hat{\alpha }}_{MLE}^{-c}=\frac{\left(n-1\right)n{\hat{\beta }}_{MLE}^{2}}{\left(n{\hat{\beta }}_{MLE}+c+1\right)\left(\left(n-1\right){\hat{\beta }}_{MLE}-c\right)}{\hat{\alpha }}_{MLE}. \end{array}$$

When *α* is unknown, we have

$$\begin{array}{cc} \int_{0}^{+\infty }\beta^{c+1}\pi \left(\beta |x\right)d\beta & =\frac{{\left(q_{2}\left(x\right)-n\log q_{1}\left(x\right)\right)}^{n}}{\tau (n)}\int_{0}^{+\infty }\beta^{n+c}\exp\left(-\left(q_{2}\left(x\right)-n\log q_{1}\left(x\right)\right)\beta \right)d\beta \\ \; & =\frac{n+c}{q_{2}\left(x\right)-n\log q_{1}\left(x\right)}\int_{0}^{+\infty }\beta^{c}\pi \left(\beta |x\right)d\beta . \end{array}$$

Thus, by Proposition 3, we have

$$\begin{array}{c} {\hat{\beta }}_{B_{c}}=\left(1+\frac{c}{n}\right){\hat{\beta }}_{MLE}. \end{array}$$

And when $c_{0}=n\left(\frac{\beta_{0}}{{\hat{\beta }}_{MLE}}-1\right)$, we have ${\hat{\beta }}_{B_{c_{0}}}=\beta_{0}$.  □

**Remark** **1.** *When β is unknown, we have*

(26) $$\begin{array}{c} {\hat{\alpha }}_{B_{c}}=\frac{1-\left(c+1\right)\int_{0}^{+\infty }\exp\left(-(c+1)u\right){\left(1+u{\hat{\beta }}_{MLE}\right)}^{-n}du}{1-c\int_{0}^{+\infty }\exp\left(-cu\right){\left(1+u{\hat{\beta }}_{MLE}\right)}^{-n}du}{\hat{\alpha }}_{MLE}. \end{array}$$

*In particular, when c=0, we get*

(27) $$\begin{array}{c} {\hat{\alpha }}_{B_{c}}={\hat{\alpha }}_{E}=\left(1-\int_{0}^{+\infty }\exp\left(-u\right){\left(1+u{\hat{\beta }}_{MLE}\right)}^{-n}du\right){\hat{\alpha }}_{MLE}. \end{array}$$

*From (27), we know that ${\hat{\alpha }}_{E}<{\hat{\alpha }}_{MLE}$. By analyzing (26), when c>0 gradually increases, ${\hat{\alpha }}_{B_{c}}$ will increase firstly and then decrease, and finally ${\hat{\alpha }}_{B_{c}}$ will converge to ${\hat{\alpha }}_{MLE}$. Let $\alpha_{0}$ be the real value of scale parameter α, then when ${\hat{\alpha }}_{E}\leq \alpha_{0}\leq {\hat{\alpha }}_{MLE}$, there exists $c_{0}\neq 0$ such that ${\hat{\alpha }}_{B_{c_{0}}}=\alpha_{0}$, When $\alpha_{0}>{\hat{\alpha }}_{E}$, ${\hat{\alpha }}_{E}$ is the closest estimation among all ${\hat{\alpha }}_{B_{c}}$. When $\alpha_{0}>{\hat{\alpha }}_{MLE}$, there exists $c_{0}\neq 0$ such that ${\hat{\alpha }}_{c_{0}}$ is the closest estimation among all ${\hat{\alpha }}_{B_{c}}$.*

**Theorem** **5.** *Using Al-Bayyati’s loss function, we find that when β is known, ${\hat{\alpha }}_{B_{c}}\left(x|\beta \right)=\frac{n\beta +c}{n\beta +c+1}{\hat{\alpha }}_{MLE}$. When α is known, ${\hat{\beta }}_{B_{c}}\left(x|\alpha \right)=\frac{n+1+c}{q_{2}\left(x\right)-n\log\;\alpha }$. In both cases, there exist $c_{1}$ and $c_{2}$ such that ${\hat{\alpha }}_{B_{c_{1}}}\left(x|\beta \right)=\alpha_{0}$ and ${\hat{\beta }}_{B_{c_{2}}}\left(x|\alpha \right)=\beta_{0}$, where $\alpha_{0}$ and $\beta_{0}$ are the real value of scale and shape parameter respectively.*

**Proof.**  When *β* is known, we have

$$\begin{array}{c} \int_{0}^{{\hat{\alpha }}_{MLE}}\alpha^{c+1}\pi \left(\alpha |x,\beta \right)d\alpha =\int_{0}^{{\hat{\alpha }}_{MLE}}t^{c+1}\mathrm{dPr}\left(\alpha \leq t|x,\beta \right)=\frac{n\beta }{n\beta +c+1}{\hat{\alpha }}_{MLE}^{c+1} \end{array}$$

and

$$\begin{array}{c} \int_{0}^{{\hat{\alpha }}_{MLE}}\alpha^{c}\pi \left(\alpha |x,\beta \right)d\alpha =\frac{n\beta }{n\beta +c}{\hat{\alpha }}_{MLE}^{c}. \end{array}$$

Then by Proposition 3, we have

$$\begin{array}{c} {\hat{\alpha }}_{B_{c}}\left(x|\beta \right)=\frac{\int_{0}^{{\hat{\alpha }}_{MLE}}\alpha^{c+1}\pi \left(\alpha |x,\beta \right)d\alpha }{\int_{0}^{{\hat{\alpha }}_{MLE}}\alpha^{c}\pi \left(\alpha |x,\beta \right)d\alpha }=\frac{n\beta +c}{n\beta +c+1}{\hat{\alpha }}_{MLE}. \end{array}$$

When *α* is known, we get

$$\begin{array}{cc} \int_{0}^{+\infty }\beta^{c+1}\pi \left(\beta |x,\alpha \right)d\beta & =\frac{{\left(q_{2}\left(x\right)-n\log\alpha \right)}^{n+1}}{\tau (n+1)}\int_{0}^{+\infty }\beta^{n+c+1}\exp\left(-(q_{2}\left(x\right)-n\log\alpha )\beta \right)d\beta \\ \; & =\frac{n+c+1}{q_{2}\left(x\right)-n\log q_{1}\left(x\right)}\int_{0}^{+\infty }\beta^{c}\pi \left(\beta |x,\alpha \right)d\beta . \end{array}$$

Thus by Proposition 3, we get

$$\begin{array}{c} {\hat{\beta }}_{B_{c}}\left(x|\alpha \right)=\frac{n+1+c}{q_{2}\left(x\right)-n\log\alpha }. \end{array}$$

When $\beta =\beta_{0}$, we have

$$\begin{array}{c} {\hat{\alpha }}_{B_{c}}\left(x|\beta_{0}\right)=\frac{n\beta_{0}+c}{n\beta_{0}+c+1}{\hat{\alpha }}_{MLE} \end{array}$$

Noting that if ${\hat{\alpha }}_{MLE}\neq \alpha_{0}$, then ${\hat{\alpha }}_{B_{c_{0}}}\left(x|\beta_{0}\right)=\alpha_{0}$, where

$$\begin{array}{c} c_{0}=\frac{\alpha_{0}}{{\hat{\alpha }}_{MLE}-\alpha_{0}}-n\beta_{0}. \end{array}$$

Hence either ${\hat{\alpha }}_{MLE}$ or ${\hat{\alpha }}_{B_{c_{0}}}\left(x|\beta_{0}\right)$ is the true value of α.

When $\alpha =\alpha_{0}$, we have

$$\begin{array}{c} {\hat{\beta }}_{B_{c}}\left(x|\alpha_{0}\right)=\frac{n+1+c}{q_{2}\left(x\right)-n\log\alpha_{0}}. \end{array}$$

Hence we can take

$$\begin{array}{c} c_{0}=\left(q_{2}\left(x\right)-n\log\alpha_{0}\right)\alpha_{0}-\left(n+1\right), \end{array}$$

such that ${\hat{\beta }}_{B_{c_{0}}}\left(x|\alpha_{0}\right)$ is the true value of *β*.  □

### 4.4. Bayesian Posterior Prediction

Let $\tilde{X}∼\pi \left(\tilde{x};\alpha ,\beta \right)$ be the value that needs to be observed from Pareto distribution. In the sense of posterior distribution (13), if the sample X is given, we can make relevant posterior prediction of $\tilde{X}$. The discussion will be divided into the following three cases.

1. When neither α nor β are unknown, then we have
  $$\begin{array}{cc} m(\tilde{x}|x) & =\int_{0}^{+\infty }\int_{0}^{+\infty }\pi \left(\tilde{x};\alpha ,\beta \right)\pi \left(\alpha ,\beta |x\right)d\alpha d\beta \\ \; & =-\int_{0}^{+\infty }\int_{0}^{+\infty }\frac{n{\left(q_{2}\left(x\right)-n\log q_{1}\left(x\right)\right)}^{n}}{\tau (n)}\beta^{n}\alpha^{n\beta -1}\exp\left(-q_{2}\left(x\right)\beta \right)I_{\left[0\leq \alpha \leq q_{1}\left(x\right)\right]}\cdot \frac{\beta \alpha^{\beta }}{{\tilde{x}}^{\beta +1}}I_{\left[\tilde{x}\geq \alpha \right]}d\alpha d\beta \\ \; & =-\int_{0}^{+\infty }\frac{n{\left(q_{2}\left(x\right)-n\log q_{1}\left(x\right)\right)}^{n}}{\tau (n)}\exp\left(-q_{2}\left(x\right)\beta \right)\frac{\beta^{n+1}}{{\tilde{x}}^{\beta +1}}d\beta \int_{0}^{\min\left(\tilde{x},q_{1}\left(x\right)\right)}\alpha^{(n+1)\beta -1}d\alpha \\ \; & =-\int_{0}^{+\infty }\frac{n{\left(q_{2}\left(x\right)-n\log q_{1}\left(x\right)\right)}^{n}}{\tau (n)}\exp\left(-q_{2}\left(x\right)\beta \right)\frac{\beta^{n+1}}{{\tilde{x}}^{\beta +1}}\cdot \frac{{\left(\min\left(\tilde{x},q_{1}\left(x\right)\right)\right)}^{(n+1)\beta }}{(n+1)\beta }d\beta \\ \; & =-\int_{0}^{+\infty }\frac{n{\left(q_{2}\left(x\right)-n\log q_{1}\left(x\right)\right)}^{n}}{\tau (n)}\beta^{n}\frac{\exp\left(-\left(-\left(n+1\right)\log\min\left(\tilde{x},q_{1}\left(x\right)\right)+\log\tilde{x}+q_{2}\left(x\right)\right)\beta \right)}{\left(n+1\right)\tilde{x}}d\beta \\ \; & =\frac{n^{2}{\left(q_{2}\left(x\right)-n\log q_{1}\left(x\right)\right)}^{n}}{\left(n+1\right)\tilde{x}{\left(-\left(n+1\right)\log\left(\min\left(\tilde{x},q_{1}\left(x\right)\right)\right)+\log\tilde{x}+q_{2}\left(x\right)\right)}^{n+1}}I_{\left[\tilde{x}>0\right]}\\ \; & =\frac{n^{2}{\left(q_{2}\left(x\right)-n\log q_{1}\left(x\right)\right)}^{n}}{\left(n+1\right)\tilde{x}}N\left(x,\tilde{x}\right), \end{array}$$
  where
  $$\begin{array}{c} N\left(x,\tilde{x}\right)=\left\{\begin{array}{cc} {\left(q_{2}\left(x\right)-n\log\tilde{x}\right)}^{-(n+1)}, & 0<\tilde{x}<{\hat{\alpha }}_{MLE}\left(x\right)\\ {\left(\left(n+1\right)\log q_{1}\left(x\right)+\log\tilde{x}+q_{2}\left(x\right)\right)}^{-(n+1)}, & \tilde{x}\geq {\hat{\alpha }}_{MLE}\left(x\right). \end{array}\right. \end{array}$$
2. When *α* is known and β is unknown, we have
  $$\begin{array}{cc} m(\tilde{x}|x,\alpha ) & =\int_{0}^{+\infty }\pi \left(\tilde{x};\alpha ,\beta \right)\pi \left(\beta |x,\alpha \right)d\beta =\frac{\left(n+1\right){\left(q_{2}\left(x\right)-n\log\alpha \right)}^{n+1}}{\tilde{x}{\left(-\left(n+1\right)\log\alpha +\log\tilde{x}+q_{2}\left(x\right)\right)}^{n+2}}I_{\left[\tilde{x}>\alpha \right]}. \end{array}$$
3. When β is known and α is unknown, we have
  $$\begin{array}{cc} m(\tilde{x}|x,\beta ) & =\int_{0}^{+\infty }\pi \left(\tilde{x};\alpha ,\beta \right)\pi \left(\alpha |x,\beta \right)d\alpha \\ \; & =\frac{n}{n+1}\beta {\hat{\alpha }}_{MLE}^{-n\beta }\left(x\right)\cdot {\tilde{x}}^{-\beta -1}{\left(\min\left(\tilde{x},{\hat{\alpha }}_{MLE}\left(x\right)\right)\right)}^{(n+1)\beta }I_{\left[\tilde{x}>0\right]}\\ \; & =\frac{n}{n+1}\beta \cdot \left\{\begin{array}{cc} {\hat{\alpha }}_{MLE}^{-n\beta }\left(x\right)\cdot {\tilde{x}}^{n\beta -1}, & 0<\tilde{x}<{\hat{\alpha }}_{MLE}\left(x\right)\\ {\hat{\alpha }}_{MLE}^{\beta }\left(x\right)\cdot {\tilde{x}}^{-\beta -1}, & \tilde{x}\geq {\hat{\alpha }}_{MLE}\left(x\right). \end{array}\right. \end{array}$$

For the above posterior prediction distribution, given the prediction credibility *k*, we can make Bayesian prediction inference in practical applications. The specific process is as follows. From $\int_{{\tilde{x}}_{L}}^{{\tilde{x}}_{U}}m\left(\tilde{x}|x\right)d\tilde{x}=k$, multiple sets of ${\tilde{x}}_{L}$, ${\tilde{x}}_{U}$ can be got. By choosing appropriately the upper and lower bounds for $\tilde{X}$ such that ${\tilde{x}}_{U}-{\tilde{x}}_{L}$ is smaller, then we can obtain higher prediction accuracy.

## 5. Simulation

In real life, the proposed algorithm for target detection of maritime radar needs to be tested and verified on sea clutter data. In order to determine the sea clutter better, it is often necessary to estimate the parameters of sea clutter model.

Therefore, in this section, we will use the conclusion of Section 4 to estimate the parameters of Pareto model of sea clutter and show the simulation results.

### 5.1. The Influence of Parameters on Sea Clutter

In this subsection, we show the effect of scale parameter *α* and shape parameter *β* on sea clutter.

**Figure 1:**
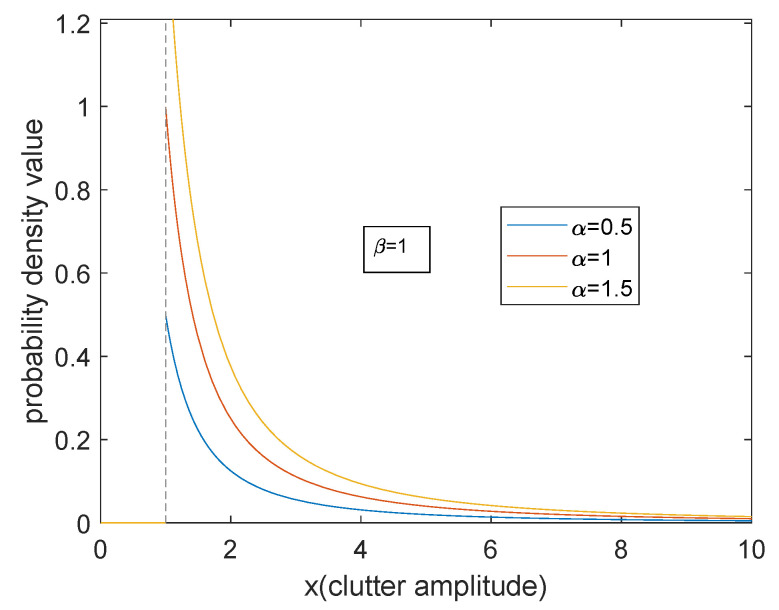
The influence of the change of α probability distributions.

**Figure 2:**
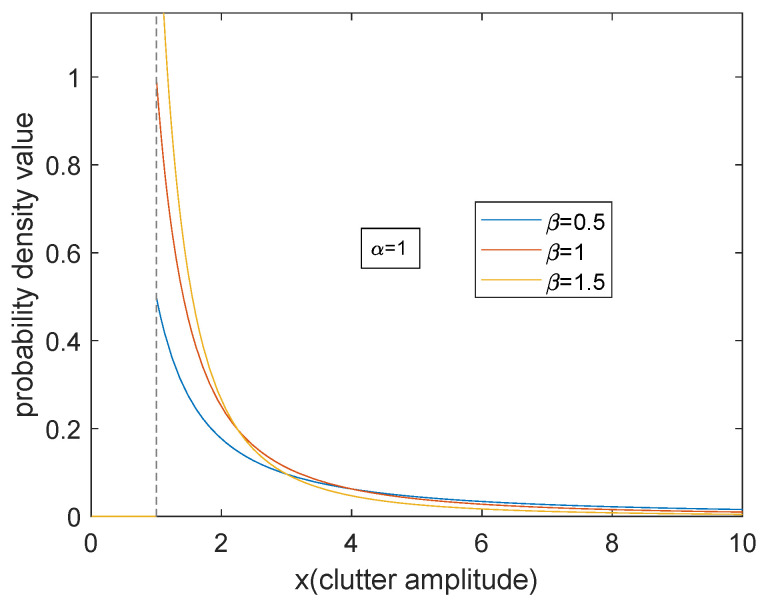
The influence of the change of β on probability distributions.

Figure 1 and Figure 2 show the probability density curve of Pareto distribution with respect to two parameters. It can be seen from the figures that when the scale parameter *α* is larger, the density curve is even. The proportion of small clutter amplitude increases, and the decline of the whole curve is gentle. As the shape parameter *β* becomes larger, the proportion of small clutter amplitude increases significantly and becomes more concentrated, and the tail descends faster. On the whole, for Pareto model, the energy is concentrated on the small clutter. The trailing phenomenon is apparent. The essential reason is that when the radar is grazing incident, the overall backscatter echo is relatively weak.

### 5.2. Various Types of Bayesian Estimation on Sea Clutter Models

In this subsection, we show the aforementioned Bayesian estimations of sea clutter.

**Figure 3:**
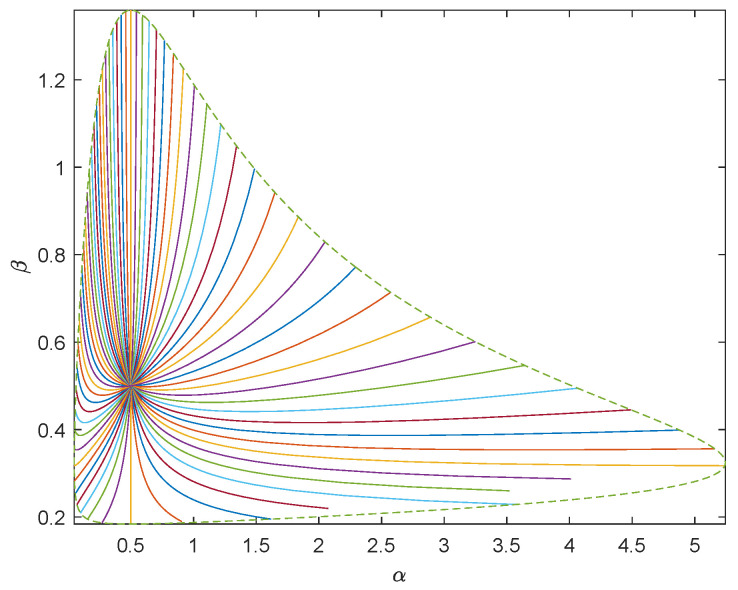
Sixty-four uniformly distributed geodesics centered on (0.5,0.5).

**Figure 4:**
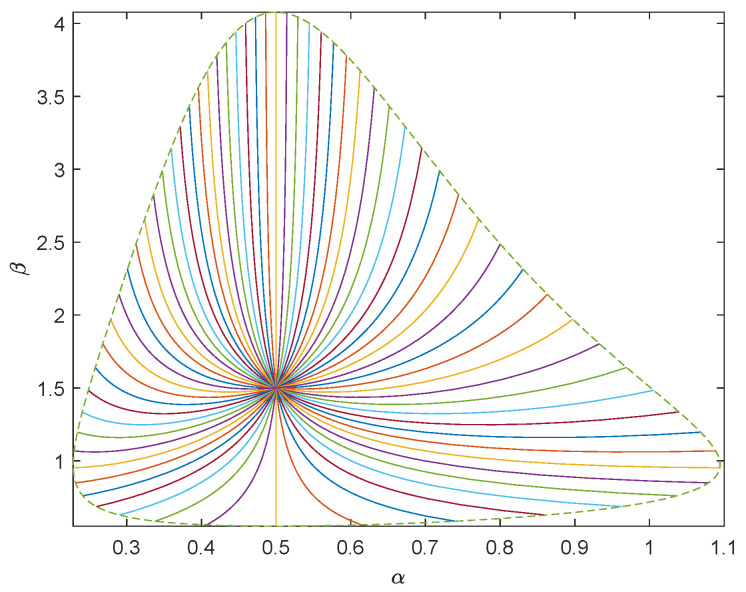
Sixty-four uniformly distributed geodesics centered on (0.5,1.5).

In order to generate random samples of Pareto distribution with parameters $\alpha_{0}$ and $\beta_{0}$, we use the inverse distribution function and take the inverse transformation method to extract Pareto samples: $X=\frac{\alpha_{0}U^{-1}}{\beta_{0}}$, where *U* is the uniformly distributed random variable on [0,1]. We carry out numerical simulations where $\alpha_{0}=0.5,1,1.5$ and $\beta_{0}=0.5,1,1.5$, respectively. Using the inverse transformation method, we generate 1000 random samples subject to Pareto distribution. To show the geometry of Pareto model of sea clutter, we take $\left(\alpha_{0},\beta_{0}\right)=\left(0.5,1\pm 0.5\right)$ as the center and draw the unit geodesic circumference with dotted line. We draw 64 uniformly distributed geodesics with directions $\theta_{0}=\frac{k\pi }{32},k=0,1,\cdots ,63$ with solid line. See Figure 3 and Figure 4.

To describe the proximity between the estimated values of each group and the predetermined parameter value $\left(\alpha_{0},\beta_{0}\right)$, we need to calculate the geodesic distance $d\{\left(\alpha_{0},\beta_{0}\right),$$\left(\hat{\alpha }\left(x\right),\hat{\beta }\left(x\right))\right\}$. If the distance between the estimated value and the predetermined parameter value is close, we believe that the estimation is accurate. By (22), $d\left(\left(\alpha_{0},\beta_{0}\right),\left(\hat{\alpha }\left(x\right),\hat{\beta }\left(x\right)\right)\right)\propto 1+\frac{\beta_{0}\hat{\beta }\left(x\right){\left(\log\alpha_{0}-\log\hat{\alpha }\left(x\right)\right)}^{2}}{2}+\frac{{\left(\beta_{0}-\hat{\beta }\left(x\right)\right)}^{2}}{2\beta_{0}\hat{\beta }\left(x\right)}$. Hence the smaller $|\log\alpha_{0}-\log\hat{\alpha }|$ and $|\beta_{0}-\hat{\beta }|$ are, the more accurate the estimation is.

Next we will make a comparative analysis of various types of Bayesian estimations.

#### 5.2.1. Mean Geodesic Estimation and the Common Bayesian Estimations

**Case 1.** Both scale parameter α and shape parameter β are unknown.

From Table 1 we know that $|{\hat{\alpha }}_{E}-{\hat{\alpha }}_{MGE}|$ and $|{\hat{\beta }}_{E}-{\hat{\beta }}_{MGE}|$ are less than $10^{-4}$, hence ${\hat{\alpha }}_{E}$ and ${\hat{\alpha }}_{MGE}$ are almost equal. Since ${\hat{\alpha }}_{E}$ does not have explicit expression, ${\hat{\alpha }}_{MGE}$ can take the place of ${\hat{\alpha }}_{E}$ and it also has more precise geometric explanation.

In most simulation tests, $\left({\hat{\alpha }}_{MGE},{\hat{\alpha }}_{MGE}\right)$ is more accurate than $\left({\hat{\alpha }}_{MLE},{\hat{\alpha }}_{MLE}\right)$ and $\left({\hat{\alpha }}_{ME},{\hat{\alpha }}_{ME}\right)$. Hence, in general the estimation MGE that we proposed is better than the common Baysesian estimations.

**Case 2.** Either shape parameter β or scale parameter α is known.

When one parameter is known, the statistical manifold is degenerated. Hence taking the Euclidean distance or the geodesic distance does not make much difference. This can be seen in Table 2 and Table 3.

Comparing Table 2 and Table 3, when β is known, the accuracy of the estimations is $10^{-3}$ and when α is known, the accuracy of all kinds of estimations is $10^{-2}$. Therefore, the accuracy of various types of estimations will improve if β is known. This indicates that scale parameter α is more easily obtained from samples in the sea clutter model and has strong robustness. Shape parameter β is more sensitive than scale parameter α.

#### 5.2.2. The Estimations under Al-Bayyati’s Loss Function

In this subsection, we give the simulation of various types of Bayesian estimations when $\left(\alpha_{0},\beta_{0}\right)=\left(1.5,1.5\right)$.

**Case 1.** Both scale parameter α and shape parameter β are unknown.

**Figure 5:**
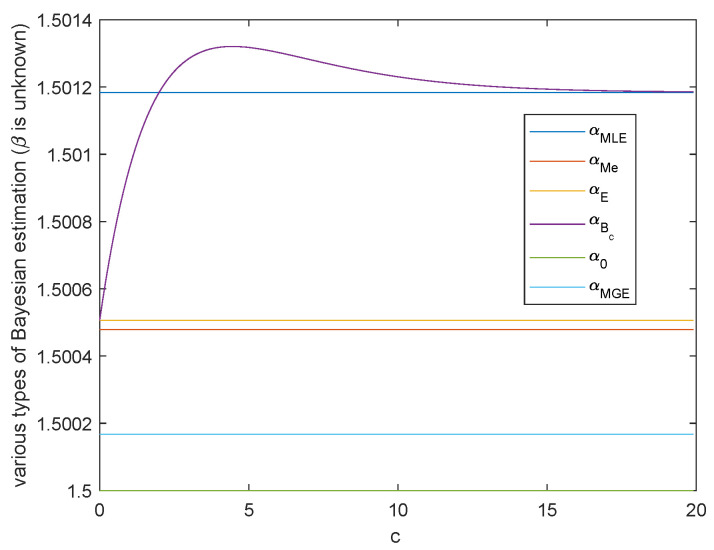
The variation of various Bayesian estimations of α with Al-Bayyati’s loss function parameter *c*.

**Figure 6:**
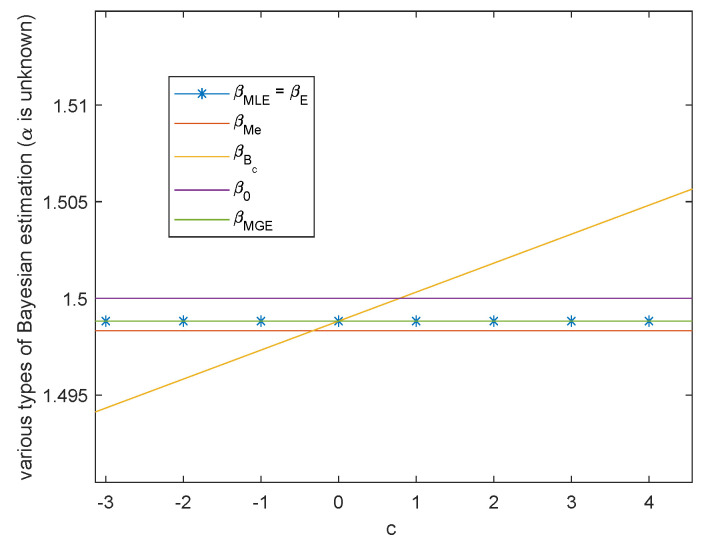
The variation of various Bayesian estimations of β with Al-Bayyati’s loss function parameter *c*.

When *α* and *β* are unknown, the variation trend of Bayesian estimation of the two parameters under Al-Bayyati’s loss function with parameter *c* is shown in Figure 5 and Figure 6, respectively. When *β* is unknown, by (26)

$${\hat{\alpha }}_{B_{c}}=\frac{1-\left(c+1\right)\int_{0}^{+\infty }\exp\left(-(c+1)u\right){\left(1+u{\hat{\beta }}_{MLE}\right)}^{-n}du}{1-c\int_{0}^{+\infty }\exp\left(-cu\right){\left(1+u{\hat{\beta }}_{MLE}\right)}^{-n}du}{\hat{\alpha }}_{MLE}.$$

Figure 5 shows the case when ${\hat{\alpha }}_{E}\geq \alpha_{0}$. Hence from the discussion in Remark 1, ${\hat{\alpha }}_{E}$ is the closest estimate among all ${\hat{\alpha }}_{B_{c}}$ with the increasing of positive *c*.

When α is unknown, by Theorem 4

$${\hat{\beta }}_{B_{c}}=\left(1+\frac{c}{n}\right){\hat{\beta }}_{MLE}.$$

When c=0, ${\hat{\beta }}_{B_{c}}={\hat{\beta }}_{MLE}={\hat{\beta }}_{E}$ and when $c_{0}=n\left(\frac{\beta_{0}}{{\hat{\beta }}_{MLE}}-1\right)$, ${\hat{\beta }}_{B_{c_{0}}}=\beta_{0}$. As shown in Figure 6, there always exists infinitely many *c* such that ${\hat{\beta }}_{c}$ is closer than the common Bayesian estimations. And $c_{0}=n\left(\frac{\beta_{0}}{{\hat{\beta }}_{MLE}}-1\right)$ is nothing but the real value of parameter β. These two figures also show that MGE are better than the common Bayesian estimations when both of the parameters are unknown. Therefore, when α and β are unknown, to obtain closer estimations, we are able to change $c_{1}$ and $c_{2}$ to make $|\log\alpha_{0}-\log{\hat{\alpha }}_{c_{1}}|$ and $|\beta_{0}-{\hat{\beta }}_{c_{2}}|$ smaller and even obtain the minimum value. Through the previous discussions, the choice of the best $c_{1}$ depends on the inequality among real parameter $\alpha_{0}$, ${\hat{\alpha }}_{MLE}$ and ${\hat{\alpha }}_{E}$. The best $c_{2}$ is $c_{2}=n\left(\frac{\beta_{0}}{{\hat{\beta }}_{MLE}}-1\right)$.

**Case 2.** Either shape parameter *β* or scale parameter *α* is known.

**Figure 7:**
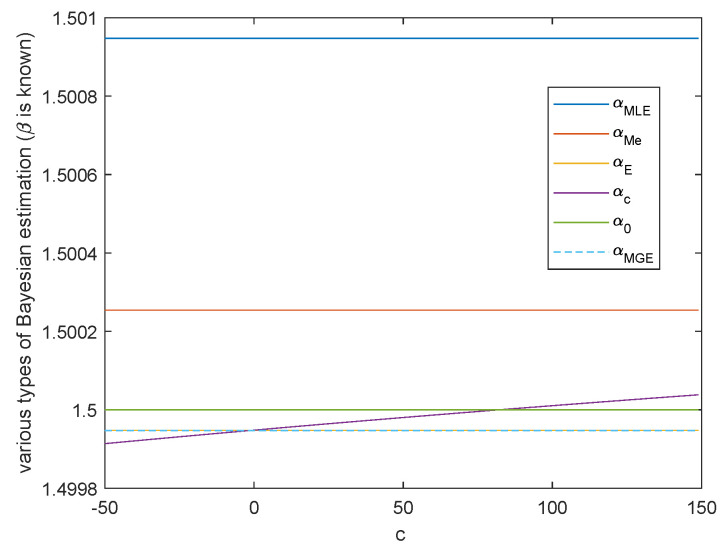
The variation of various Bayesian estimations of α with loss function parameter *c* when $\beta =\beta_{0}$.

**Figure 8:**
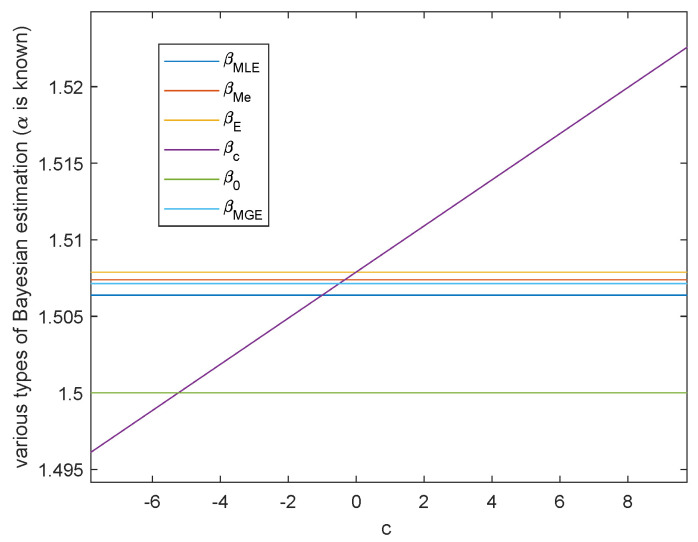
The variation of various Bayesian estimations of β with loss function parameter *c* when $\alpha =\alpha_{0}$.

When β or α is known, the variation trend of various Bayesian estimation of parameter α or parameter β in Al-Bayyati’s loss function with parameter *c* is shown in Figure 7 or Figure 8, respectively. When $\beta =\beta_{0}$, by Theorem 5, we get

$$\begin{array}{c} {\hat{\alpha }}_{B_{c}}\left(x|\beta \right)=\frac{n\beta +c}{n\beta +c+1}{\hat{\alpha }}_{MLE}. \end{array}$$

If ${\hat{\alpha }}_{MLE}\neq \alpha_{0}$, we have ${\hat{\alpha }}_{B_{c}}\left(x|\beta \right)=\alpha_{0}$ when $c=\frac{\alpha_{0}}{{\hat{\alpha }}_{MLE}-\alpha_{0}}-n\beta$. Hence either ${\hat{\alpha }}_{MLE}$ is the true value of α, or we can take $c_{0}=\frac{\alpha_{0}}{{\hat{\alpha }}_{MLE}-\alpha_{0}}-n\beta$ such that ${\hat{\alpha }}_{B_{c_{0}}}\left(x|\beta \right)$, which is the true value of α. This is shown in Figure 7.

When $\alpha =\alpha_{0}$, by Theorem 5, we get

$${\hat{\beta }}_{B_{c}}\left(x|\alpha \right)=\frac{n+1+c}{q_{2}\left(x\right)-n\log\alpha }.$$

If $c=\left(q_{2}\left(x\right)-n\log\;\alpha \right)\alpha_{0}-\left(n+1\right)$, then ${\hat{\beta }}_{B_{c}}\left(x|\alpha \right)=\beta_{0}$. Hence we can take $c_{0}=\left(q_{2}\left(x\right)-n\log\alpha \right)\alpha_{0}-\left(n+1\right)$ such that ${\hat{\beta }}_{B_{c_{0}}}\left(x|\alpha \right)$ is the true value of β. This is shown in Figure 8.

In above two cases, there are infinitely many *c* such that ${\hat{\alpha }}_{c}\left(x|\beta \right)$ or ${\hat{\alpha }}_{c}\left(x|\alpha \right)$ is closer than the common Bayesian estimations.

### 5.3. Simulation of Posterior Predictive Distribution

**Figure 9:**
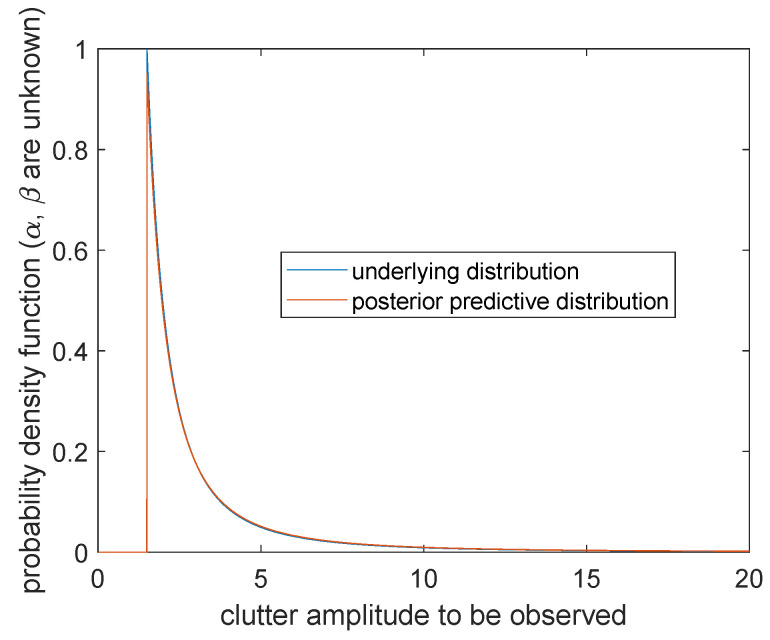
Posterior predictive distribution and underlying distribution (α and β are unknown).

**Figure 10:**
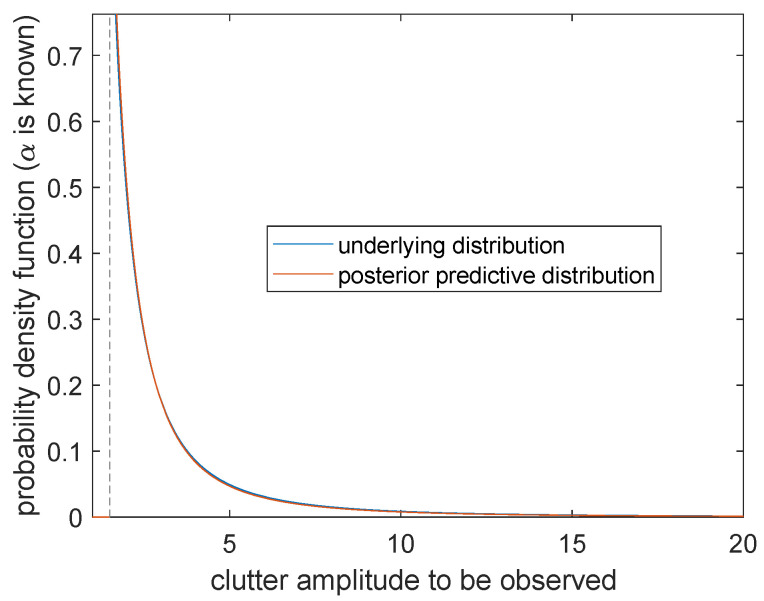
Posterior predictive distribution and underlying distribution ($\alpha =\alpha_{0}$).

**Figure 11:**
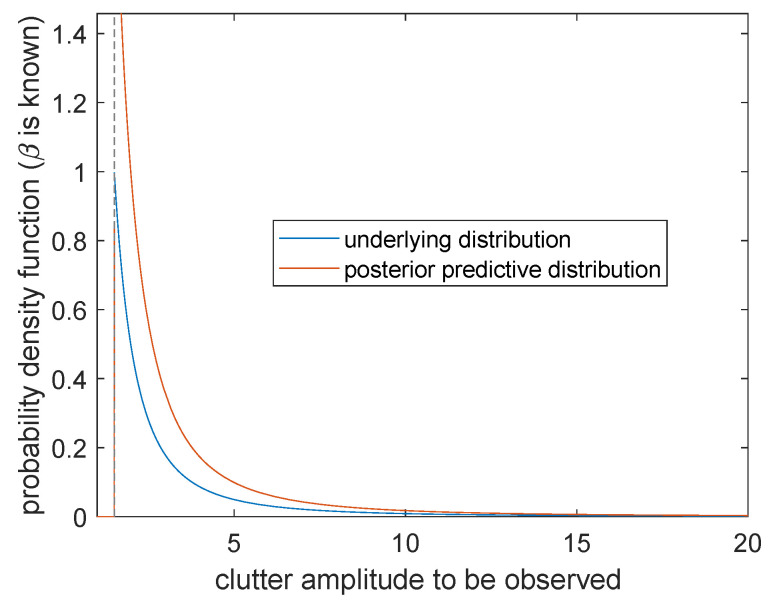
Posterior predictive distribution and underlying distribution ($\beta =\beta_{0}$).

In order to observe the simulation effect of the posterior prediction distribution, according to the samples generated in Section 5.2, we drew the posterior prediction distribution of sea clutter and the real Pareto distribution $\pi (x|\alpha_{0},\beta_{0})$ of sea clutter where $\left(\alpha_{0},\beta_{0}\right)=\left(1.5,1.5\right)$ for comparative analysis. See Figure 9, Figure 10 and Figure 11.

**Case 1.** Scale parameter *α* and shape parameter *β* are unknown

The posterior prediction distribution of sea clutter is

(28) $$m\left(\tilde{x}|x\right)=\frac{n^{2}{\left(q_{2}\left(x\right)-n\log q_{1}\left(x\right)\right)}^{n}}{\left(n+1\right)\tilde{x}}N\left(x,\tilde{x}\right),$$

where

$$N\left(x,\tilde{x}\right)=\left\{\begin{array}{cc} {\left(q_{2}\left(x\right)-n\log\tilde{x}\right)}^{-(n+1)}, & 0<\tilde{x}<{\hat{\alpha }}_{MLE}\left(x\right)\\ {\left(-\left(n+1\right)\log q_{1}\left(x\right)+\log\tilde{x}+q_{2}\left(x\right)\right)}^{-(n+1)}, & \tilde{x}\geq {\hat{\alpha }}_{MLE}\left(x\right). \end{array}\right.$$

The image is shown in Figure 9. The blue curve represents the probability distribution of sea clutter $\pi (x|\alpha_{0},\beta_{0})$, which gives positive values at the right side of the boundary point $x=\alpha_{0}$. The orange curve represents the posterior prediction distribution of sea clutter $m(\tilde{x}|x)$, which changes continuously when $\tilde{x}>0$, but forms a cusp at $\tilde{x}={\hat{\alpha }}_{MLE}$. Compared with the two curves, the curve of the predicted distribution of sea clutter is connected by a continuous curve and shifts slightly to the left. It is worth noting that although $m(\tilde{x}|x)$ tends to infinity as $\tilde{x}\to 0^{+}$, it is not reflected in the image and can be ignored in the actual calculation of the probability.

**Case 2.***α* is known and *β* is unknown

The posterior prediction distribution of sea clutter is

(29) $$m\left(\tilde{x}|x,\alpha \right)=\frac{\left(n+1\right){\left(q_{2}\left(x\right)-n\log\alpha \right)}^{n+1}}{\tilde{x}{\left(\left(n+1\right)\log\alpha +\log\tilde{x}+q_{2}\left(x\right)\right)}^{n+2}}I_{\left[\tilde{x}>\alpha \right]}.$$

It can be seen from Figure 10 that the probability distribution $\pi (x|\alpha_{0},\beta_{0})$ (the blue curve) and the posterior prediction distribution $m(\tilde{x}|x,\beta )$ (the orange curve) can only obtain positive values at the right side of the boundary point $x=\alpha_{0}$. There is a very high degree of overlap, which means that when α is known, the prediction is going to be very accurate. We come to the conclusion that more effective information can be obtained for parameter α than β.

**Case 3.***β* is known and *α* is unknown

The posterior prediction distribution of sea clutter is

(30) $$m\left(\tilde{x}|x,\beta \right)=\frac{n}{n+1}\beta \cdot \left\{\begin{array}{cc} {\hat{\alpha }}_{MLE}^{-n\beta }\left(x\right)\cdot {\tilde{x}}^{n\beta -1}, & 0<\tilde{x}<{\hat{\alpha }}_{MLE}\left(x\right)\\ {\hat{\alpha }}_{MLE}^{\beta }\left(x\right)\cdot {\tilde{x}}^{-\beta -1}, & \tilde{x}\geq {\hat{\alpha }}_{MLE}\left(x\right). \end{array}\right.$$

The probability distribution $\pi (x|\alpha_{0},\beta_{0})$ (the blue curve) obtains a positive value at the right side of boundary point $x=\alpha_{0}$. The posterior prediction distribution $m(\tilde{x}|x,\alpha )$ (the orange curve) changes continuously at $\tilde{x}>0$ and forms a cusp at $\tilde{x}={\hat{\alpha }}_{MLE}$. By comparing these two curves, the posterior prediction distribution shows a significant right shift, and the simulation effect is not ideal near $\tilde{x}={\hat{\alpha }}_{MLE}$. However, with the continuous increasing of $\tilde{x}$, the two curves gradually coincide and the prediction accuracy becomes higher. Therefore, when β is known and α is unknown, the larger the clutter amplitude is to be observed, the higher the prediction accuracy will be.

To sum up, for the sea clutter model, the Bayesian posterior prediction results under the above three conditions are ideal, and the prediction model can well reflect the characteristics of sea clutter towing.

## 6. Conclusions and Future Work

In this paper, we presented systematic methods for Bayesian inference from geometric viewpoints and applied it to Pareto model. We carried out simulations on sea clutter to show the effectiveness.

For Pareto model, there does not exist general *α*-parallel prior. Using the Jeffreys prior and by using geodesic distance and Al-Bayyati’s loss function, we obtain two new classes of Bayesian estimations. We call the estimation in the sense of mean geodesic distance MGE and it is proved that MGE has following advantages: it has the explicit expression, and is more accurate than the common Bayesian estimations which has shown in our simulation. We also prove that the estimations under the Al-Bayyati’s loss function are more accurate than the common Bayesian estimations. Actually, there are infinitely many *c* such that the new estimations are better. These results are important for the estimation of parameters when studying sea clutter model. Finally we show that the Bayesian posterior prediction results can well reflect the characteristics of sea clutter towing in any case.

In the future, more in-depth researches are worth discussing. From statistical viewpoints, we can apply Bayesian inference for the Pareto model to non-linear regression models [20]. From geometrical viewpoints, we expect to generalize our framework and combine more tools from information geometry. We want to carry out more experiments and applications in different fields.

## Figures and Tables

**Table 1:** Mean geodesic estimations and the common Bayesian estimations (α,β are not known).

$\left(\alpha ,\beta \right)$	$\left({\hat{\alpha }}_{\mathrm{MLE}},{\hat{\beta }}_{\mathrm{MLE}}\right)$	$\left({\hat{\alpha }}_{\mathrm{Me}},{\hat{\beta }}_{\mathrm{Me}}\right)$	$\left({\hat{\alpha }}_{E},{\hat{\beta }}_{E}\right)$	$\left({\hat{\alpha }}_{\mathrm{MGE}},{\hat{\beta }}_{\mathrm{MGE}}\right)$
(0.5,0.5)	(0.5024,0.5171)	(0.5017,0.5169)	(0.5014,0.5171)	(0.5014,0.5171)
(0.5,1.0)	(0.5001,1.0292)	(0.4998,1.0288)	(0.4997,1.0292)	(0.4997,1.0292)
(0.5,1.5)	(0.5003,1.5566)	(0.5001,1.5561)	(0.5000,1.5566)	(0.5000,1.5566)
(1.0,0.5)	(1.0053,0.4743)	(1.0038,0.4742)	(1.0032,0.4743)	(1.0032,0.4743)
(1.0,1.0)	(1.0009,1.0209)	(1.0002,1.0205)	(0.9999,1.0209)	(0.9999,1.0209)
(1.0,1.5)	(1.0001,1.4639)	(0.9996,1.4634)	(0.9994,1.4639)	(0.9994,1.4639)
(1.5,0.5)	(1.5033,0.4883)	(1.5012,0.4881)	(1.5002,0.4883)	(1.5002,0.4883)
(1.5,1.0)	(1.5003,1.0226)	(1.4993,1.0223)	(1.4988,1.0226)	(1.4988,1.0226)
(1.5,1.5)	(1.5010,1.4978)	(1.5003,1.4973)	(1.5003,1.4978)	(1.5000,1.4978)

**Table 2:** Mean geodesic estimations and the common Bayesian estimations (β is known).

$\beta =\beta_{0}$	${\hat{\alpha }}_{\mathrm{Me}}\left(x|\beta_{0}\right)$	${\hat{\alpha }}_{E}\left(x|\beta_{0}\right)$	${\hat{\alpha }}_{E}\left(x|\beta_{0}\right)$	${\hat{\alpha }}_{\mathrm{MGE}}\left(x|\beta_{0}\right)$
(0.5,0.5)	0.5024	0.5017	0.5014	0.5014
(0.5,1.0)	0.5001	0.4998	0.4996	0.4996
(0.5,1.5)	0.5003	0.5000	0.4999	0.4999
(1.0,0.5)	1.0053	1.0039	1.0033	1.0033
(1.0,0.5)	1.0009	1.0002	0.9999	0.9999
(1.0,1.5)	1.0001	0.9996	0.9994	0.9994
(1.5,0.5)	1.5033	1.5012	1.5003	1.5003
(1.5,1.0)	1.5003	1.4992	1.4988	1.4988
(1.5,1.5)	1.5010	1.5003	1.5000	1.5000

**Table 3:** Mean geodesic estimations and the common Bayesian estimations (*α* is known).

$\alpha =\alpha_{0}$	${\hat{\beta }}_{\mathrm{MLE}}\left(x|\alpha_{0}\right)$	${\hat{\beta }}_{\mathrm{Me}}\left(x|\alpha_{0}\right)$	${\hat{\beta }}_{E}\left(x|\alpha_{0}\right)$	${\hat{\beta }}_{\mathrm{MGE}}\left(x|\alpha_{0}\right)$
(0.5,0.5)	0.5158	0.5162	0.5163	0.5161
(0.5,1.0)	1.0289	1.0295	1.0299	1.0294
(0.5,1.5)	1.5553	1.5563	1.5568	1.5560
(1.0,0.5)	0.4732	0.4735	0.4736	0.4734
(1.0,0.5)	1.0199	1.0206	1.0210	1.0205
(1.0,1.5)	1.4638	1.4647	1.4652	1.4645
(1.5,0.5)	0.4877	0.4881	0.4882	0.4880
(1.5,1.0)	1.0224	1.0231	1.0234	1.0229
(1.5,1.5)	1.4921	1.4931	1.4936	1.4928

## Data Availability

Data is synthetically generated and described in the paper.
